# Dissociable Neural Systems Underwrite Logical Reasoning in the Context of Induced Emotions with Positive and Negative Valence

**DOI:** 10.3389/fnhum.2014.00736

**Published:** 2014-09-23

**Authors:** Kathleen W. Smith, Oshin Vartanian, Vinod Goel

**Affiliations:** ^1^York University, Toronto, ON, Canada; ^2^University of Toronto Scarborough, Toronto, ON, Canada; ^3^University of Hull, Hull, UK; ^4^IRCCS Fondazione Ospedale San Camillo, Venice, Italy

**Keywords:** reasoning, emotion, fMRI, IAPS, belief-bias, positive, negative

## Abstract

How emotions influence syllogistic reasoning is not well understood. fMRI was employed to investigate the effects of induced positive or negative emotion on syllogistic reasoning. Specifically, on a trial-by-trial basis participants were exposed to a positive, negative, or neutral picture, immediately prior to engagement in a reasoning task. After viewing and rating the valence and intensity of each picture, participants indicated by keypress whether or not the conclusion of the syllogism followed logically from the premises. The content of all syllogisms was neutral, and the influence of belief-bias was controlled for in the study design. Emotion did not affect reasoning performance, although there was a trend in the expected direction based on accuracy rates for the positive (63%) and negative (64%) versus neutral (70%) condition. Nevertheless, exposure to positive and negative pictures led to dissociable patterns of neural activation during reasoning. Therefore, the neural basis of deductive reasoning differs as a function of the valence of the context.

## Introduction

Although the empirical literature examining the effects of emotion on cognition is very large, relatively few studies have investigated the effect of emotion on logical reasoning. Behavioral studies that have investigated this effect have usually found that compared to neutral valence, positive and negative valence result in impaired accuracy in logical reasoning. This has been shown to be true regardless of whether the emotions are manipulated via the content of the logical arguments (Lefford, 1946), mood of the participants (Melton, 1995; Oaksford et al., 1996), or both (Blanchette and Richards, 2004; Blanchette, 2006). See also the review by Blanchette and Richards (2010).

However, other studies have reported no impairment in cognitive processing associated with negative emotion. In fact, sadness and depression have been found to promote systematic cognitive processing (Alloy and Abramson, 1979; Schwarz and Bless, 1991; Bless et al., 1992; Bohner et al., 1992; Edwards and Weary, 1993). Blanchette et al. (2007) found that reasoning in the negative condition improved logical reasoning by reducing belief-bias, but only when the material referred to participants’ actual exposure to terrorist activity; otherwise, reasoning in the negative condition was impaired, both for other participant groups on all negative material and for the group exposed to actual terrorist activity on non-terror-related negative material. Goel and Vartanian (2011) found that, when argument logic and beliefs about the material itself required opposite responses (incongruence) on a given trial, reasoning performance was better when the reasoning material was politically incorrect than when otherwise. These results suggest that under some conditions negative content can improve reasoning performance.

The inconsistency in the literature on the effect of emotion on cognitive processes could arise from various sources, such as variations in the type of stimulus materials, incongruence between argument logic and one’s beliefs about the content, or presentation of the emotion as either part of the content or separately, as part of the context.

To extend this literature, we explored whether the effects of emotion on underlying reasoning processes differ depending on whether the emotion is positive or negative. This exploration was motivated by evidence suggesting that positive and negative emotions may exert different effects on cognition. Positive emotion promotes creativity (Isen et al., 1987) and facilitates noticing more relations among concepts (Isen and Daubman, 1984). It also promotes a reliance on such heuristic shortcuts as source expertise and stereotyping instead of considering the evidence when making evaluations (Schwarz and Clore, 1983; Bless et al., 1992; Bodenhausen et al., 1994). Positive emotion also impairs working memory (Martin and Kerns, 2011), and distracts attention toward task-irrelevant information (Biss and Hasher, 2011) at the level of early sensory encoding (Vanlessen et al., 2013). The bulk of available evidence suggests that positive emotion might exert its deleterious effects on reasoning by taxing working memory with induced bottom-up task-irrelevant information and by promoting a top-down heuristic processing mode.

There is now good evidence to suggest that positive and negative emotion induction have different effects on the brain. Using a gender identification task (to reduce attention to the emotion manipulation), Schmitz et al. (2009) found that positive emotion broadened focus to peripherally presented stimuli (houses) and was accompanied by neural activation in right lateral frontal pole (BA 10), lateral orbitofrontal cortex (BA 11), as well as by correlated activity in parahippocampal place area and primary visual cortex. In contrast, negative emotion narrowed focus to targets (faces) only, and was accompanied by neural activation in amygdala, as well as by inversely correlated activity in parahippocampal place area and primary visual cortex. In Schmitz et al. (2009), emotion had been induced by means of pictures from the International Affective Picture System (IAPS; Lang et al., 1997). In Dolcos et al. (2004), valence ratings of positive and negative IAPS pictures during scanning were accompanied by different patterns of neural activation; positive evaluations were associated with activation in left dorsolateral prefrontal cortex (BA 8/9), whereas negative evaluations were associated with activation in bilateral dorsolateral prefrontal cortex (BA 8/9) and right ventrolateral prefrontal cortex (BA 47). Using only negative IAPS pictures, Taylor et al. (2000) found that activation in the amygdala, uncus, and anterior parahippocampal gyrus was positively correlated with increasingly aversive ratings of pictures; as well, mildly aversive ratings were associated with activation in left-hemisphere posterior and subcortical regions, whereas strongly aversive ratings were associated with activation in bilateral posterior and subcortical regions and lateral orbitofrontal cortex. In general, the above reports suggest that, apart from activation in orbitofrontal cortex, positive and negative emotion induction lead to differentiated underlying patterns of neural activity; positive emotion is accompanied by medial frontal and left frontal activation, whereas negative emotion is accompanied by activation in amygdala and bilateral or right frontal activation. Patterns of activation in posterior cortical and in subcortical regions (apart from amygdala) vary depending on the task but, within these studies, differ by valence or intensity of emotion.

In the first neuroimaging study to examine the effect of emotion on deductive reasoning, Goel and Dolan (2003b) demonstrated that reasoning with negatively charged material was associated with activation in ventromedial prefrontal cortex, whereas reasoning with neutral material was associated with activation in left dorsolateral prefrontal cortex; furthermore, these neural mechanisms were activated in a reciprocal manner. In that study, emotion was manipulated using the content of the syllogism such that, depending on the condition, content was either emotionally provocative or neutral. The results demonstrated that the pattern of neural activation during reasoning varies as a function of emotional content.

In the present study, we sought to extend the findings of Goel and Dolan (2003b) by making an important change to the paradigm. Whereas Goel and Dolan varied the emotionality of the content itself, we chose to manipulate the emotionality of the context in which reasoning about neutral material would take place. Specifically, on each trial, participants first viewed and rated a picture on valence and intensity, and after the picture was removed from view, they engaged in a syllogistic reasoning task involving visually presented syllogisms with non-emotional content. This design feature enabled us to analyze the neural correlates of reasoning separately from those acquired during emotion induction itself. Secondly, whereas the emotional content in Goel and Dolan was negative and provocative, in the current study, we chose to induce not only negative but also positive emotion.

Therefore, the current study utilized a 3 (Emotion) × 2 (Task) within-subjects design, where the three levels of the Emotion factor were positive, neutral, and negative, and the two levels of the Task factor were reasoning and baseline. Also, because it is known that reasoning is subject to a belief-bias effect (Evans, 2003), we controlled for belief-bias in the study design.

Because of the more common findings in the literature, that is, that reasoning is impaired by positive or negative emotion manipulation, we hypothesized that each of positive and negative emotion would be detrimental to reasoning. Additionally, we hypothesized that the neural systems underlying reasoning under those two conditions would differ from that in the neutral condition.

## Materials and Methods

### Participants

Data were acquired from 16 participants (7 males, 9 females). Education levels ranged from partially completed undergraduate study to completed graduate degrees, with a mean of 17.54 (SD = 3.82) years of education. Ages ranged from 19 to 56 (mean age was 28, SD = 10 years). All participants gave informed consent. The study was approved by the York University Research Human Participants Ethics Committee.

### Stimuli

Pictures, normed as to emotional valence, were taken from the IAPS system (Lang et al., 1997). The valence categories from the IAPS were used to choose 40 positive and 40 negative pictures for the experiment. In addition, 40 pictures of furniture were added, to serve as neutral pictures.

Reasoning stimuli consisted of 75 syllogisms that were emotionally neutral in content. The arguments in 38 of these syllogisms were logically valid, whereas the arguments in the remaining 37 were logically invalid. An example of a valid syllogism is “All dogs are pets; All poodles are dogs; All poodles are pets,” and an example of an invalid syllogism is “All paper is absorbent; All napkins are paper; No napkins are absorbent.”

As well, there were 45 baseline “syllogisms,” in which the concluding sentence was taken from a different syllogism in the dataset, thereby ensuring that the conclusion of the baseline would be unrelated to the content of the two premises. Thus, in a baseline trial, the participant would prepare to respond to what was expected to be a syllogism; however, the unrelated conclusion would indicate that the stimulus is not an argument and can be rejected without integrating the conclusion into the premises.

### Study design

**Figure 1:**
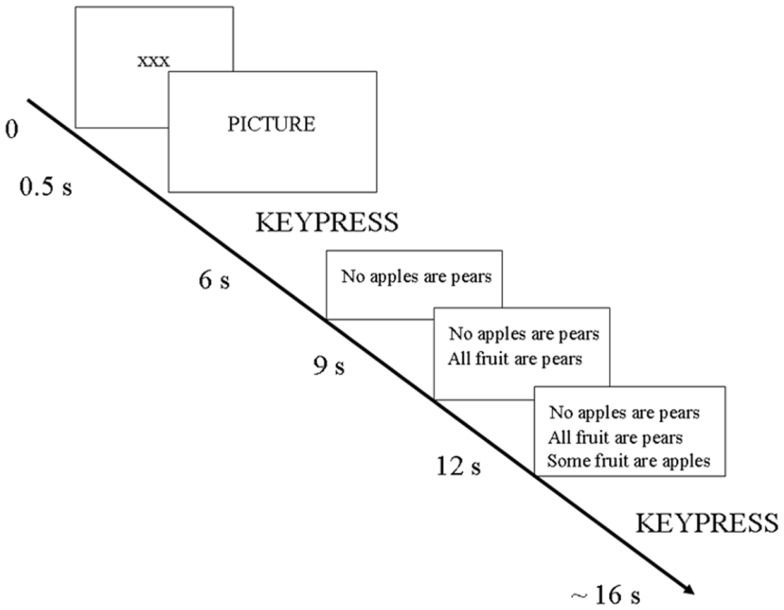
**Design of one trial**.

The study involved 120 trials delivered over 3 sessions (or “runs”) in the scanner. Each trial involved the following sequence (see Figure 1): first, the participant saw a slide with the fixation point (xxx) for 500 ms; then the fixation point disappeared. Next, the participant viewed a picture and pressed one of eight keys to indicate simultaneously the rating of positive or negative valence and the intensity of the picture’s emotional content. The specific meaning of the keys will be explained below. Then, the picture disappeared and a syllogism was presented over three consecutive slides (slide one: first premise alone; slide two: first two premises together; slide three: the two premises plus the conclusion). The syllogism remained in view during the reasoning period. The participant pressed a key to indicate whether the conclusion followed or not from the two statements (premises). Disappearance of the picture and syllogism slides was not entrained to the responses but was timed to be in synchrony with the acquisition of the brain scans. Trials varied in length and were approximately 16–20 s.

The specific meaning of the eight picture-rating keys is as follows: valence and intensity were captured in the same keypress. There were four keys in one direction for “increasingly negative” and four in the other direction for “increasingly positive.” The side was counterbalanced among participants. Participants used the index finger of each hand to respond. All participants were declared as right-handed.

The effect of belief-bias was controlled for. That is, the reasoning syllogisms were balanced overall for validity and for congruence between logic and beliefs. Congruence occurs when the argument logic is valid and the conclusion is believable or when the argument logic is invalid and the conclusion is unbelievable. Incongruence occurs when the argument logic is valid and the conclusion is unbelievable or when the argument logic is invalid and the conclusion is believable.

Thus, syllogisms and baseline trials were matched to pictures so that there were equivalent numbers of congruent syllogisms, incongruent syllogisms, and baselines within each level of the emotion factor (positive, negative, and neutral). Then the order of the 120 trials was randomized. Finally, the trials were segregated into three presentation sets of 40 trials each (see Supplementary Material). Thus, pictures were not presented in blocks by valence; the valences (positive, neutral, and negative) were quasi-randomly intermixed. The order of presentation of these three sets was counterbalanced among participants, one set for each session (“run”) in the scanner.

### fMRI scanning technique

A 1.5-T Siemens VISION system (Siemens, Erlangen, Germany) was used to acquire T1 anatomical volume images (1 mm × 1 mm × 1.5 mm voxels) and T2*-weighted images (64 × 64, 3 mm × 3 mm pixels, TE = 40 ms), obtained with a gradient echo-planar sequence using blood oxygenation level-dependent (BOLD) contrast. Echo-planar images (2 mm thick) were acquired axially every 3 mm, positioned to cover the whole brain. Each volume was partitioned into 36 slices, obtained at 90 ms per slice. Data were recorded during a single acquisition period. Volume (vol) images, 243 per session, were acquired continuously, for a total of 729 images over three sessions, with a repetition time (TR) of 3.24 s/vol. The first six volumes in each session were discarded (leaving 237 per session) to allow for T1 equilibration effects.

### Data analysis

#### Behavior

Behavioral data were analyzed using SPSS, version 16.0 (SPSS Inc., Chicago, IL, USA).

In the design there were 120 trials, 75 (62.5%) involving reasoning and 45 (37.5%) baselines. Data from two participants were discarded because of movement artifacts in the neuroimaging data. Therefore, the behavioral analyses are based on 14 participants. Twelve participants completed all three sessions of 40 trials each. One participant completed two sessions. One other participant completed all three sessions, but because some of the scan volumes were missing from the data, it was necessary to excise three trials from the middle of Session 1 and one trial from the middle of Session 2. Thus, there were a total of 12 × 120 + 80 + 116 = 1636 trials. Of these, 1021 (62.4%) were reasoning trials and 615 (37.6%) were baselines. The participants’ valence ratings were sorted into three categories: positive, negative, and neutral. Ratings of −2, −3, or −4 were classified as “negative”; ratings of +2, +3, or +4 were classified as “positive.” Ratings of −1 or +1 were considered “neutral.”

#### Neuroimaging

The functional imaging data were preprocessed and subsequently analyzed using Statistical Parametric Mapping SPM8 (Friston et al., 1994; Wellcome Department of Imaging Neuroscience; http://www.fil.ion.ucl.ac.uk/spm/).

All functional volumes were spatially realigned to the first volume. Data from two participants with head movement >2 mm were discarded. All volumes were temporally realigned to the AC–PC slice, to account for different sampling times of different slices. A mean image created from the realigned volumes was co-registered with the structural T1 volume and the structural volumes spatially normalized to the Montreal Neurological Institute brain template (Evans et al., 1993) using non-linear basis functions (Ashburner and Friston, 1999). The derived spatial transformation was then applied to the realigned T2* volumes, which were finally spatially smoothed with a 12 mm FWHM isotropic Gaussian kernel in order to make comparisons across subjects and to permit application of random field theory for corrected statistical inference (Worsley and Friston, 1995). The resulting time series across each voxel were high-pass filtered with a cut-off of 128 s, using cosine functions to remove section-specific low-frequency drifts in the BOLD signal. Global means were normalized by proportional scaling to a grand mean of 100, and the time series temporally smoothed with a canonical hemodynamic response function to swamp small temporal autocorrelations with a known filter.

Condition effects at each voxel were estimated according to the general linear model and regionally specific effects compared using linear contrasts. Each contrast produced a statistical parametric map of the *t* statistic for each voxel, which was subsequently transformed to a unit normal *Z* distribution. The BOLD signal was modeled as a canonical hemodynamic response function with time derivative. All events were modeled in the design matrix, but events of no interest (the first two sentences, and the two motor responses on a trial-by-trial basis) were modeled out. Positive, neutral, and negative picture viewing/rating were each modeled as an epoch from picture onset up to but excluding the motor response. Positive, neutral, and negative reasoning, and positive, neutral, and negative baseline were each modeled as an event. The onset of the event was the halfway point between presentation of the concluding sentence and the motor response.

Parametric (correlational) analyses were conducted to determine neural regions associated with increasingly intense positive and negative picture ratings. The BOLD signal was modeled as a canonical hemodynamic response function. All events were modeled in the design matrix, but events of no interest (the three sentences, and the two motor responses on a trial-by-trial basis) were modeled out. Positive intensity and negative intensity were each modeled as an event from picture onset.

The individual-level analyses involving emotion induction were subsequently analyzed at the group level in a random effects model, using *t*-tests (see Table 1 in Supplementary Material). The individual-level analyses of the reasoning time window were analyzed at the group level in a random effects model, using a 2 (Task: Reasoning, Baseline) × 3 Emotion (positive, negative, neutral) factorial design, with correction for non-sphericity and with proportional overall grand mean scaling (see Table 2 in Supplementary Material).

All reported results survived a threshold of *p* < 0.005 and an extent of *k* ≥ 20 voxels, a combination that has been demonstrated to produce a desirable balance between type I and type II error rates (Lieberman and Cunningham, 2009).

## Results

### Behavioral results

For each participant, we computed the proportion of each of positive:total ratings, neutral:total ratings, and negative:total ratings. For example, one participant rated 119 of the 120 trials, of which 39 were rated neutral; therefore, for this participant, the proportion of neutral:total ratings is 0.33. A repeated-measures analysis, multivariate approach, was conducted; the within-subjects factor was choice of valence (positive, neutral, and negative) and the dependent variable was mean proportion. Participants rated a significantly greater proportion of pictures as positive than as negative (*F*_2,11_ = 9.988, *p* = 0.003, partial η^2^ = 0.645).

The mean response time to rate the pictures was calculated for each participant, separately for each valence. A repeated-measures analysis, multivariate approach, was conducted; the within-subjects factor was Emotion (positive, neutral, and negative) and the dependent variable was mean picture-rating response time. Data were analyzed for 13 participants, as 1 participant had not rated any picture as “neutral.” Participants took significantly longer to rate pictures as positive than as neutral (*F*_2,11_ = 5.739, *p* = 0.02, partial η^2^ = 0.511).

The mean (SD) proportion of total picture ratings for each valence was as follows: positive 0.3859 (0.108), neutral 0.2731 (0.130), negative 0.2308 (0.085); the mean (SD) response time in milliseconds to rate the pictures was as follows: positive 2184 (483), neutral 1919 (623), negative 2092 (467). See “Behavioral Scores” in Supplementary Material.

For the reasoning trials, the overall proportion of correct:total responses was 0.630. For baselines (where the correct response would always be “not valid”), the proportion of correct:total responses was 0.972. Mean reaction time was 4185 (SD 789) ms on reasoning trials overall (that is, without regard to accuracy), and 1874 (SD 456) ms on baseline trials. This difference was significant: paired *t*(13) = 8.567, *p* = 0.001.

The proportion of correct reasoning responses to the total number of reasoning trials was computed for each participant within each valence. For instance, 1 participant rated 20 of the pictures (on reasoning trials) as positive, and reasoned logically on 15 of those trials; thus, the proportion of correct responses on positively valenced reasoning trials was 0.75 for that participant. Next, a repeated-measures analysis of variance (*n* = 13; the one participant who had not rated any pictures as neutral was excluded from this analysis), multivariate approach, was conducted to test whether the valence rating affected reasoning. The independent variable was the emotion factor (positive, neutral, and negative), and the dependent variable consisted of each participant’s mean proportion of correct:total reasoning responses. The result was not significant (*p* = 0.391, partial η^2^ = 0.157). Overall, the valence of the picture did not significantly influence subsequent reasoning. See “Behavioral Scores” in Supplementary Material.

A repeated-measures analysis of variance, multivariate approach, indicated that mean reaction time to reasoning syllogisms overall (that is, collapsed across accuracy) did not differ by Emotion (positive, neutral, and negative). Participants responded significantly more slowly on reasoning trials when their response was incorrect than when it was correct, regardless of the valence of the trial. The main effect of accuracy was significant: *F*(1, 12) = 7.537, *p* = 0.018, partial η^2^ = 0.386; there was no main effect of Emotion (positive versus negative) and no significant interaction of Accuracy × Emotion). Mean (SD) reaction times in milliseconds to syllogisms, by valence and accuracy, were as follows: for correct responses (*n* = 13), mean (SD) was 3480 (574) for positive, 3759 (729) for neutral, and 3793 (461) for negative. For incorrect responses (*n* = 9), mean (SD) was 4215 (673) for positive, 4199 (691) for neutral, and 4008 (755) for negative. For the sake of consistency with the other results, we repeated this analysis using correct trials only (repeated-measures, multivariate approach), and found that mean reaction time when responding correctly to syllogisms did not differ significantly by Emotion (*p* = 0.267, partial η^2^ = 0.213).

#### Manipulation check demonstrating the need to control for belief-bias

Instantiation of belief-bias in the current design would be as follows: on trials where there is incongruence between argument logic and beliefs (valid argument and false belief, or invalid argument and true belief), responses should be less logical and slower than on trials where there is congruence between argument logic and beliefs (valid argument and true belief, or invalid argument and false belief). We controlled for belief-bias in the study design, by ensuring equivalent numbers of congruent syllogisms, incongruent syllogisms, and baselines within each level of the emotion factor.

We thank a reviewer for suggesting that we should test directly this possible effect of belief-bias, at the behavioral level. The proportion of correct:total responses was analyzed for congruence with beliefs (congruent, incongruent) by Emotion (positive, neutral, and negative) using a repeated-measures analysis (multivariate approach). The main effect of Congruence was significant (*F*_1,12_ = 6.835, *p* = 0.023, partial η^2^ = 0.363) and the Congruence × Emotion interaction approached significance (*F*_2,11_ = 3.194, *p* = 0.081, partial η^2^ = 0.367). Thus, correct responding is significantly hindered when the logic of the argument conflicts with beliefs, tending to be more so (reduced to chance level) after positive and negative than after neutral picture ratings.

The mean proportions (SD) correct:total were as follows (*n* = 13): for congruent syllogisms, positive:total was 0.727 (0.252), neutral:total was 0.729 (0.174), and negative:total was 0.762 (0.233). For incongruent syllogisms, positive:total was 0.537 (0.174), neutral:total was 0.659 (0.267), and negative:total was 0.504 (0.305).

The mean reaction time (RT) to the syllogisms where the response was correct was analyzed for congruence with beliefs (congruent, incongruent) by Emotion (positive, neutral, and negative) using a repeated-measures analysis (multivariate approach). The main effect of Congruence was significant (*F*_1,11_ = 39.740, *p* < 0.001, partial η^2^ = 0.783); the Congruence*Emotion interaction was not significant (*p* = 0.151, partial η^2^ = 0.315). Thus, correct responses are significantly slower when the logic of the argument conflicts with beliefs, regardless of valence.

Mean reaction times (*n* = 12) when responding correctly were as follows: (a) congruent positive: 3097 ms (SD 530); (b) congruent neutral: 3437 ms (SD 532); (c) congruent negative: 3410 ms (SD 499); (d) incongruent positive: 3901 ms (SD 829); (e) incongruent neutral: 3585 ms (SD 1077); (f) incongruent negative: 4466 ms (SD 625).

### Neuroimaging results

#### Neuroimaging analysis: emotion induction time window

As indicated in Table 1 of Supplementary Material, the contrast positive–neutral yielded neural activation in left thalamus, right cerebellum, occipital lobe bilaterally, left parietal (supramarginal gyrus and secondary somatosensory area), right inferior parietal lobe, and left fusiform gyrus. The contrast negative–neutral yielded neural activation in left putamen, right amygdala, occipital lobe bilaterally, left inferior parietal (secondary somatosensory cortex and supramarginal gyrus), right inferior parietal (supramarginal gyrus), and right inferior frontal gyrus (triangularis, area 45). The contrast positive–negative yielded neural activation in left cerebellum, right hippocampus, left postcentral gyrus, and superior temporal gyrus bilaterally. The contrast negative–positive yielded neural activation in left amygdala and insula, left middle cingulate, right hippocampus, left occipital lobe, inferior parietal (supramarginal gyrus) bilaterally, left superior parietal (area 7), right precuneus, right postcentral gyrus, inferior frontal gyrus (left opercularis area 44, right area 44), left frontal (supplementary motor area and area 4), right precentral gyrus (areas 44 and 6), and superior frontal gyrus bilaterally. See Table 1 in Supplementary Material.

**Figure 2:**
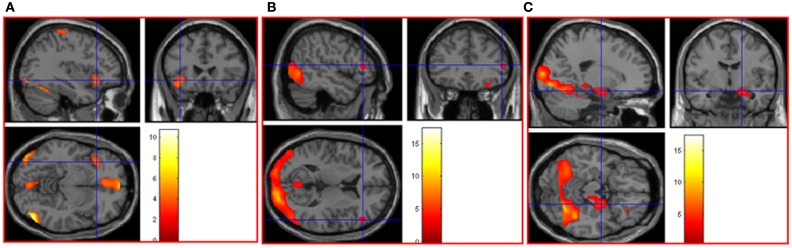
**(A)** As picture ratings increase in positive intensity, activation increases in left inferior frontal gyrus (orbitalis) (MNI co-ordinates: −36, 24, −8, *k* = 310, *Z* = 3.54) and other areas (see Table 1 in Supplementary Material). As picture ratings increase in negative intensity, activation increases in **(B)** right inferior frontal gyrus (triangularis: area 45; MNI co-ordinates: 52, 32, 10, *k* = 57, *Z* = 3.31) and in **(C)** right amygdala (MNI co-ordinates: 20, −6, −16, *k* = 744, *Z* = 3.81), as well as other areas (see Table 1 in Supplementary Material).

Parametric (correlational) analyses were conducted to determine neural regions associated with increasingly intense positive and negative picture ratings. As positive intensity increased, significant neural activation was noted in cerebellum bilaterally, left thalamus, occipital lobe bilaterally, postcentral gyrus bilaterally, middle temporal gyrus bilaterally, right inferior temporal gyrus, right fusiform gyrus, and left inferior frontal gyrus. See Table 1 in Supplementary Material and Figure 2A. As negative intensity increased, significant neural activation was noted in right amygdala, right occipital lobe, and right inferior frontal gyrus. See Table 1 in Supplementary Material and Figures 2B,C.

#### Neuroimaging analysis: reasoning time window

Neural activations associated with the reasoning time window are listed in Table 2 in Supplementary Material.

The contrast positive reasoning–positive baseline yielded neural activation in right thalamus, right occipital lobe, left parietal (supramarginal gyrus), right middle temporal gyrus, and right precentral gyrus. The contrast negative reasoning–negative baseline yielded neural activation in occipital lobe bilaterally, left inferior parietal lobe (supramarginal gyrus), left postcentral gyrus, left middle temporal gyrus, and left inferior frontal gyrus (triangularis). The contrast positive reasoning–neutral reasoning yielded activation in right inferior parietal (supramarginal gyrus). The contrast negative reasoning–neutral reasoning yielded neural activation in inferior occipital lobe bilaterally, left superior parietal lobe, left postcentral gyrus, right supramarginal gyrus, left inferior temporal and right middle temporal gyrus, left hippocampus, left middle frontal gyrus, and right frontal gyrus area 6. The contrast positive reasoning–negative reasoning yielded neural activation in left insula, right thalamus, superior temporal gyrus bilaterally, and right inferior frontal gyrus (orbitalis). The contrast negative reasoning–positive reasoning yielded significant neural activation in caudate nucleus bilaterally, left insula, occipital lobe bilaterally, left precuneus, and left postcentral gyrus.

To determine whether neural activation underlying reasoning in the positive and neutral time windows would differ after removing baseline effects, we analyzed the interaction contrast [(positive reasoning–positive baseline) − (neutral reasoning–neutral baseline)]; this analysis yielded neural activation in left middle cingulate, occipital lobe bilaterally, left inferior parietal lobe (angular gyrus), left intraparietal sulcus, right postcentral gyrus, left precentral gyrus, and right supplementary motor area.

To determine whether neural activation underlying reasoning in the negative and neutral time windows would differ after removing baseline effects, we analyzed the interaction contrast [(negative reasoning–negative baseline) − (neutral reasoning–neutral baseline)]; this analysis yielded neural activation in left superior parietal, inferior parietal lobe (angular gyrus) bilaterally, left inferior parietal (supramarginal gyrus), left postcentral gyrus, left inferior frontal gyrus (triangularis), and right supplementary motor area.

The interaction contrast [(neutral reasoning–neutral baseline) − (positive reasoning–positive baseline)] yielded neural activation in right fusiform gyrus. The interaction contrast [(neutral reasoning–neutral baseline) − (negative reasoning–negative baseline)] yielded neural activation in right hippocampus.

**Figure 3:**
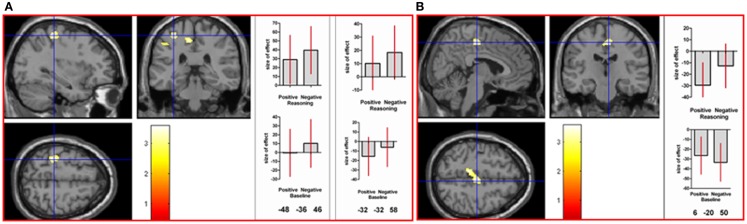
**A conjunction analysis demonstrated activation in common between the positive and negative reasoning time windows in (A) left postcentral gyrus (at the crosshair; MNI co-ordinates: −32, −32, 58, *k* = 122, *Z* = 3.43) and intraparietal sulcus (shown to the left of the crosshair in the coronal image; MNI co-ordinates: −48, −36, 46, *k* = 34, *Z* = 2.78), and in (B) right supplementary motor area (MNI co-ordinates: 6, −20, 50, *k* = 226, *Z* = 3.34), as well as other areas (see Table 2 in Supplementary Material)**. Graphs show size of effect (beta) with 5% confidence interval.

To determine areas activated in common in the positive and negative reasoning time window, we performed a conjunction analysis of two interaction contrasts: [(positive reasoning–positive baseline) − (neutral reasoning–neutral baseline)] and [(negative reasoning–negative baseline) − (neutral reasoning–neutral baseline)]. This conjunction analysis revealed neural activation in left superior parietal lobe, left inferior parietal lobe (angular gyrus, intraparietal sulcus, and supramarginal gyrus), left postcentral gyrus, and right supplementary motor area (see Figure 3).

**Figure 4:**
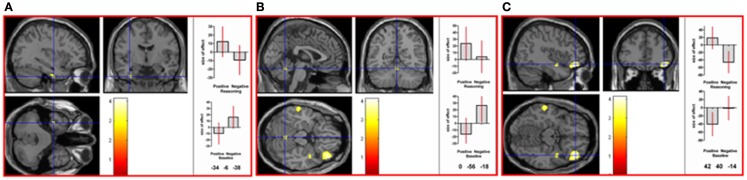
**Neural activation associated with the positive reasoning time window that is not shared with the negative reasoning time window occurs in (A) left fusiform gyrus (MNI co-ordinates: −34, −6, −38, *k* = 28, *Z* = 3.06), in (B) the vermis of the cerebellum (MNI co-ordinates: 0, −56, −18, *k* = 35, *Z* = 2.9), in (C) right inferior frontal gyrus (orbitalis; MNI co-ordinates: 42, 40, −14, *k* = 428, *Z* = 3.91), and in right superior parietal lobe (not shown) (see Table 2 in Supplementary Material)**. Graphs show size of effect (beta) with 5% confidence interval.

**Figure 5:**
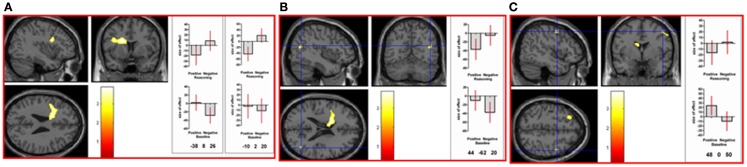
**Neural activation associated with the negative reasoning time window that is not shared with the positive reasoning time window occurs in (A) left caudate nucleus (MNI co-ordinates: −10, 2, 20, *k* = 594, *Z* = 3.39) extending into left inferior frontal gyrus (opercularis; MNI co-ordinates: −38, −8, 26, *Z* = 3.35), in (B) right middle temporal gyrus (relative deactivation; MNI co-ordinates: 44, −62, 20, *k* = 39, *Z* = 2.86), in (C) right precentral gyrus (area 6; MNI co-ordinates: 48, 0, 50, *k* = 38, *Z* = 2.85), as well as in left occipital lobe (not shown) (See Table 2 in Supplementary Material)**. Graphs show size of effect (beta) with 5% confidence interval.

To directly compare neural activations in the positive and negative reasoning time window, we conducted two interaction contrasts as follows. The interaction contrast [(positive reasoning–positive baseline) − (negative reasoning–negative baseline)] yielded neural activation in cerebellum (vermis), right superior parietal lobe, left fusiform gyrus, and right inferior frontal gyrus (orbitalis) (see Figure 4). The interaction contrast [(negative reasoning–negative baseline) − (positive reasoning–positive baseline)] yielded neural activation in left caudate nucleus, left occipital lobe, left inferior frontal gyrus (opercularis), and right precentral gyrus, as well as relative deactivation in right middle temporal gyrus (see Figure 5).

## Discussion

The above-chance reasoning accuracy levels indicate that participants were engaged in the task. The emotion manipulations were also successful, as indicated by the variation in participants’ ratings of picture valence.

### Emotion induction

Patterns of neural responses during picture viewing/rating were consistent with those reported in the literature. As positive intensity increased, activation was noted in the left inferior frontal cortex. Likewise, Dolcos et al. (2004) reported neural activation in frontal cortex, left hemisphere only, in association with the rating of positive pictures. Furthermore, there is a trend in the neuroimaging literature (Wager et al., 2003) for left-lateralization in the frontal lobe associated with approach-related emotions^1^.

During negative picture viewing/rating, activations in the contrast (negative picture–neutral picture) included right amygdala and right inferior frontal gyrus. Activations in the contrast (negative picture–positive picture) included left amygdala and inferior frontal gyrus bilaterally. As negative intensity increased, activations were in right occipital, right amygdala, and right inferior frontal gyrus. In Dolcos et al. (2004), rating of negative pictures was associated with neural activation in bilateral frontal regions. In Taylor et al. (2000), ratings of aversiveness of negative pictures were associated with neural activation in amygdala, uncus, and anterior parahippocampus. Neuroimaging studies of emotion perception (including studies using the IAPS) often report activation in amygdala, parahippocampal cortex, pregenual anterior cingulate, dorsal inferior frontal gyrus, inferior temporal and occipital cortex, and lateral cerebellum (Wager et al., 2008); withdrawal-related emotions^2^ are generally correlated with bilateral frontal activation (Murphy et al., 2003) and with amygdala activation (Wager et al., 2003).

### Reasoning

Based on existing literature, we had hypothesized that both positive and negative emotion would be detrimental to subsequent reasoning. We did not find a significant difference in either reasoning accuracy or mean reaction time among the positive, neutral, and negative conditions. The Congruence*Emotion manipulation check indicated that reasoning was impaired when beliefs and logic were incongruent; however, we did not have the power to explore this at the neural level, because of design choices we made at the outset. Further study of this issue may be warranted (see Supplementary Material).

There have been other studies showing that emotion does not necessarily impair reasoning. Specifically, negative emotions have not invariably been associated in the literature with impaired reasoning. Goel and Vartanian (2011) conducted a behavioral study in which they manipulated the conflict between argument logic and beliefs about the conclusion by introducing politically incorrect material; on incongruent trials (a valid argument with an unbelievable conclusion, or an invalid argument with a believable conclusion), reasoning performance was better when the statement was politically incorrect than when otherwise. Blanchette et al. (2007) found that reasoning in the negative condition (compared to neutral) improved only when the reasoning material was related to participants’ actual exposure to terrorist activity, whereas reasoning about other negative material was impaired.

Blanchette and Leese (2011) found no relation between reasoning performance and participant ratings of the intensity of negative and neutral stimuli. It is intriguing to note a similarity between their study and ours; Blanchette and Leese’s study may be the first to link deductive reasoning with physiological arousal (measured with transient skin conductance response) underlying negative emotion induction, and ours may be the first study using pictures from the IAPS to link deductive reasoning with neural activation (measured using fMRI) underlying positive and negative emotion induction. Blanchette and Leese found no relation between reasoning performance and participant ratings of the intensity of negative and neutral stimuli, whereas our study found no effect on reasoning performance of positive or negative emotion induction in a design that included participant ratings.

Our main interest, reflected in our hypotheses, was to show that the neural systems underlying reasoning in each of the positive and negative conditions would differ from those in the neutral condition. These hypotheses were supported.

First, results indicated a crossover interaction, or double dissociation, between the positive and neutral reasoning time windows at the neural level. Not only did the interaction contrast [(positive reasoning–positive baseline) − (neutral reasoning–neutral baseline)] reveal activations but so also did the reverse interaction contrast [(neutral reasoning–neutral baseline) − (positive reasoning–positive baseline)]. Thus, although reasoning after positive emotion induction is not impaired, it is implemented at the neural level differently than is neutral reasoning. The neural pattern associated with the positive reasoning time window involves increased activation in left middle cingulate, occipital lobes bilaterally, left inferior parietal (angular gyrus), left intraparietal sulcus, right postcentral gyrus, left precentral gyrus, and right supplementary motor area.

A double dissociation indicates those neural regions implicated in condition A but not in condition B, and simultaneously, those neural regions implicated in condition B but not in condition A. Therefore, it indicates that conditions A and B involve separable systems.

Activation in the left inferior parietal lobe has been associated with abstract reasoning (Goel et al., 2000; Goel, 2009; Kuo et al., 2009; Watson and Chatterjee, 2012). Activation in the left angular gyrus has been associated with semantic meaning (Seghier et al., 2010; Sharp et al., 2010), more so when there is a conflict involving implausible sentences (Ye and Zhou, 2009) or when the stimulus is emotional (Hervé et al., 2012); it is implicated also in problem identification (Dandan et al., 2013b), in problem solving (Dandan et al., 2013a; Grabner et al., 2013), and in cognitive flexibility (Jacobson et al., 2011). Activation in intraparietal sulcus has been associated with item-specific processing but not with relations among items (Ackerman and Courtney, 2012), with symbolic number processing (Bugden et al., 2012), with attention to items presented in the periphery (Gillebert et al., 2013), and with temporal orienting (that is, attention toward a specific moment in time; Davranche et al., 2011). Left frontal precentral gyrus has been associated with the interaction of attention and language comprehension (Kristensen et al., 2013), with syntax complexity and *post hoc* reanalysis of sentence comprehension (Meltzer et al., 2010), and with successful inhibitory control (Padmala and Pessoa, 2010). Activation in postcentral gyrus has been associated with the illusory perception of motion (Planetta and Servos, 2012), and with visceral stimulation (Hojo et al., 2012; Kaplan and Meyer, 2012). The right frontal supplementary motor area has been associated with speeded decision-making (Wenzlaff et al., 2011), with attention maintenance (Kristensen et al., 2013), and is considered to be part of a ventral attention network that mediates bottom-up capture of attention by memory (Burianová et al., 2012).

Secondly, results indicated a crossover interaction, or double dissociation, between the negative and neutral reasoning time windows at the neural level. Not only did the interaction contrast [(negative reasoning–negative baseline) − (neutral reasoning–neutral baseline)] reveal activations but so also did the reverse interaction contrast [(neutral reasoning–neutral baseline) − (negative reasoning–negative baseline)]. Thus, although reasoning after negative emotion induction is not impaired, it is implemented at the neural level differently than is neutral reasoning. The neural pattern associated with the negative reasoning time window involves left postcentral gyrus, left inferior parietal (supramarginal gyrus), left superior parietal lobe, inferior parietal (angular gyrus) bilaterally, left inferior frontal gyrus, and right supplementary motor area.

As mentioned above, activation in postcentral gyrus has been associated with the illusory perception of motion and with visceral stimulation. Left supramarginal gyrus is considered to be part of a ventral attention network (Corbetta et al., 2008) that mediates bottom-up capture of attention by memory (Burianová et al., 2012). Superior parietal lobe is involved in the interaction between language processing and the control of movement (Segal and Petrides, 2012); activation has been associated with syllogistic reasoning involving abstract or incongruent materials (Tsujii et al., 2011). As mentioned above, activation in the left inferior parietal lobe has been associated with abstract reasoning; activation in the left angular gyrus has been associated with semantic meaning, more so when there is a conflict involving implausible sentences or when the stimulus is emotional, with problem identification and problem solving, and with cognitive flexibility. Activation in the left inferior frontal region has been associated with semantic integration (Yu et al., 2011; Huang et al., 2012) and with categorization (Lupyan et al., 2012; Philipp et al., 2013). As mentioned above, activation in the right supplementary motor area has been associated with speeded decision-making and with attention maintenance, and is considered to be part of a ventral attention network that mediates bottom-up capture of attention by memory.

The positive and negative reasoning time windows yielded similar activation in left superior parietal, left inferior parietal (angular gyrus, intraparietal sulcus, and supramarginal gyrus), left postcentral gyrus, and right supplementary motor area. This finding emerged from a conjunction analysis of two interaction contrasts: [(positive reasoning–positive baseline) − (neutral reasoning–neutral baseline)] and [(negative reasoning–negative baseline) − (neutral reasoning–neutral baseline)].

Beyond these similarities, however, results indicated a crossover interaction, or double dissociation, between the positive and negative reasoning time windows at the neural level. Not only did the interaction contrast [(positive reasoning–positive baseline) − (negative reasoning–negative baseline)] reveal activations but so also did the reverse interaction contrast [(negative reasoning–negative baseline) − (positive reasoning–positive baseline)].

The interaction favoring the positive reasoning time window revealed activation in right inferior frontal (orbitalis, or BA 47), right superior parietal, cerebellar vermis, and left fusiform. In the literature, activation in right frontal (BA 47) has been noted in unconstrained hypothesis generation (Vartanian and Goel, 2005). As mentioned above, superior parietal lobe is involved in the interaction between language processing and the control of movement. The cerebellar vermis is involved in autonomic and motor responses to an emotional state (Strata et al., 2011). Activation in left fusiform has been involved in lexico-semantic processing (Tsapkini and Rapp, 2010; Thesen et al., 2012).

The interaction favoring the negative reasoning time window revealed activation in left caudate nucleus, left inferior frontal (opercularis, or BA 44), left occipital lobe, and right precentral gyrus, as well as relative deactivation in right middle temporal gyrus. In the literature, caudate nucleus has been shown to have a crucial role in reasoning (Melrose et al., 2007) unless insufficient processing time has been allotted for reasoning (Kalbfleisch et al., 2007). Activation in left inferior frontal (BA 44) is associated more with phonological than with semantic fluency (Katzev et al., 2013). Right precentral gyrus is implicated in the representation of coordinated hand–mouth movements (Desmurget et al., 2014) and the neural coding of oculomotor and somatomotor space (Iacoboni et al., 1997). Activation in right middle temporal lobe has been associated with verbal fluency (Krug et al., 2011) and with semantic priming (Laufer et al., 2011).

Goel and Dolan (2003b) had manipulated emotion using the content of the syllogism such that content was either emotionally provocative or neutral; they found that reasoning with negatively charged material was associated with activation in ventromedial prefrontal cortex, whereas reasoning with neutral material was associated with activation in left dorsolateral prefrontal cortex. We have extended their findings by manipulating emotion separately from the material itself. Our emotion manipulation provides an emotional context in which to reason about neutral material, rather than providing emotional content. Therefore, it is not surprising that our findings differ from those in Goel and Dolan (2003b). Reasoning in an emotional but unrelated context involves a different neural underpinning than does reasoning about emotional content.

The fact that we found neural level differences in reasoning, despite a lack of behavioral difference, suggests that the neural systems underlying reasoning are sensitive to neural systems previously recruited by emotional context, and can to some extent compensate for these effects of emotions. It is possible that the behavioral manifestations (that is, impairment of reasoning) emerge only when the system is stressed.

In summary, we had predicted that both positive and negative emotion would be detrimental to reasoning, and that the neural systems underlying reasoning under those two conditions would differ from that in the neutral condition. We found that, although neither positive nor negative emotional context significantly impaired reasoning performance, positive and negative context did have dissociable effects on the underlying neural mechanisms involved in reasoning.

## Conflict of Interest Statement

The authors declare that the research was conducted in the absence of any commercial or financial relationships that could be construed as a potential conflict of interest.

## Supplementary Material

The Supplementary Material for this article can be found online at http://www.frontiersin.org/Journal/10.3389/fnhum.2014.00736/abstract

## Footnotes

^1^Approach emotions include anger but are otherwise positive; none of our stimuli were designed to induce anger.

^2^Withdrawal emotions are negative in valence.
