# Supplementary material for: Dissociable Neural Systems Underwrite Logical Reasoning in the Context of Induced Emotions with Positive and Negative Valence
Source: Front Hum Neurosci. 2014 Sep 23;8:736. doi: 10.3389/fnhum.2014.00736 (PMC4172059; doi:10.3389/fnhum.2014.00736)
Supplement: Supplementary file 1 [file Data_Sheet_1.DOC]

*Supplementary Material*

Dissociable neural systems underwrite logical reasoning in the context of induced emotions with positive and negative valence

Kathleen W. Smith^1^, Oshin Vartanian^2^, and Vinod Goel^1,3,4^*

^1^York University, Toronto, Canada

^2^University of Toronto—Scarborough, Toronto, Canada.

^3^University of Hull, UK

^4^IRCCS Fondazione Ospedale San Camillo, Lido, Venice, Italy

**Behavioral Scores**

**Behav 1.1** As stated in section 2.5.1, there were 1636 trials, across the dataset, available for analysis.

The number of these trials by participant valence-rating is as follows:

Positive Neutral Negative No rating

700 426 382 128

A chi-square test of goodness of fit (with ‘no rating’ excluded) indicated that the number of pictures differed significantly by valence-rating from expected (where ‘expected’ was equal numbers per valence; χ^2^ (*_df_* _= 3)_ = 118.127, *p* < .001).

The proportion of these trials by participant valence-rating is as follows:

Positive Neutral Negative No rating

0.4279 0.2604 0.2335 0.0782

**Behav 1.2** For analysis of the reasoning data, we excluded baseline trials. Therefore, as stated in section 2.5.1, there were 1021 reasoning trials across the dataset, available for analysis. All syllogism material was neutral. The number of reasoning trials across the dataset, by condition (based on the participant valence-ratings of the picture in the same trial), is as follows:

Condition

Positive Neutral Negative No rating

415 264 261 81

A chi-square test of goodness of fit (with ‘no rating’ excluded) indicated that the number of reasoning trials differed significantly by condition from expected (where ‘expected’ was equal numbers per condition; χ^2^ (*_df_* _= 2)_ = 49.496, *p* < .001).

The proportion of all reasoning trials by condition is as follows:

Positive Neutral Negative No rating

0.4065 0.2586 0.2556 0.0793

**Behav 1.3** All of the above results pertain to calculations across the dataset.

**Behav 2.1** As described in section 3.1, we computed the proportion of [each condition]:total picture ratings within each participant’s data. We then computed the *mean* proportion of [each condition]:total picture ratings. These means were based on *n* = 13 as one participant did not rate any picture as neutral. The results are as follows:

Mean (*SD*) proportion of picture ratings by valence (*n* = 13):

Positive Neutral Negative No rating

0.3859 0.2731 0.2308 0.1102

(calculated as ‘1 – sum of the other means’;

8 of 14 participants rated every picture)

(0.108) (0.130) (0.085)

A repeated-measures analysis, multivariate approach, was conducted; the within-subjects factor was choice of valence (positive, neutral, negative; we excluded ‘no rating’ from this analysis) and the dependent variable was mean proportion. Participants rated a significantly greater proportion of pictures as positive than as negative (*F_2,11_*  = 9.988, *p* = .003, partial η^2^ = .645).

**Behav 2.2** As described in section 3.1, we computed the proportion of [each condition]:total (of all) reasoning trials within each participant’s data. We then computed the *mean* proportion of [each condition]:total (of all) reasoning trials. These means were based on *n* = 13 as one participant did not have any trials in the neutral condition (because that participant had not rated any of the pictures as ‘neutral’, and ‘condition’ is based on participant ratings of the picture on the same trial as the syllogism). The results are as follows:

Mean (*SD*) proportion of reasoning trials by condition (*n* = 13):

Positive Neutral Negative No rating

0.3823 0.2797 0.2549 0.0831

(0.109) (0.127) (0.091) (0.142)

A repeated-measures analysis, multivariate approach, was conducted; the within-subjects factor was reasoning condition (positive, neutral, negative; we excluded ‘no rating’ from this analysis) and the dependent variable was mean proportion. The proportion of reasoning trials was significantly greater in the positive than in the negative condition (*F_2,11_*  = 4.589, *p* = .036, partial η^2^ = .455).

**Behav 3.1** The next set of results pertain to only those reasoning trials where the response was logical (correct).

As described in section 3.1, we computed the proportion of [each condition]:total *correct* reasoning trials within each participant’s data. We then computed the *mean* proportion of [each condition]:total correct reasoning trials. These means were based on *n* = 13 as one participant did not have any trials in the neutral condition (as explained above). The results are as follows:

Mean (*SD*) proportion of reasoning trials by condition (*n* = 13):

Positive Neutral Negative No rating

0.6285 0.7010 0.6440 0.6546 (*n* = 8)

(0.166) (0.165) (0.224) (0.298)

A repeated-measures analysis, multivariate approach, was conducted; the within-subjects factor was ‘correct reasoning’ condition (positive, neutral, negative; we excluded ‘no rating’ from this analysis) and the dependent variable was mean proportion. The proportion of ‘correct reasoning’ trials did not differ significantly by condition (*p* = .391, partial η^2^ = .157).

**Behav 4.1** How these data are utilized:

section explanation

- 1. How many pictures there were (altogether) by valence (Ps’ ratings). See the next point.

1.2 How many reasoning trials were available altogether, by condition (based on

Ps’ picture ratings on the same trial), for data analysis. This is a design issue,

as we wanted to ensure that we were measuring the actual effect of the

emotion manipulation (on ‘subsequent reasoning’ neural activation), rather

than neural activation based on normed ratings. Note that we had included

sufficient trials at the outset to ensure that we would still have enough trials in

each reasoning condition after Ps exercised this choice.

1.3 ‘Across the dataset’ is a reference to # of trials, not organized by participant

- 1. Ps were free to choose picture valence and this section shows

that, on average, Ps rated more pictures as positive than as negative.

- 1. A direct consequence of the above. On average, Ps had more reasoning

trials in the positive than in the negative condition. For the neural analyses of

the reasoning timewindow, these trials were utilized. At the first (individual)

level of neural analysis, one contrast image is generated for each P for each

condition; thus, for each P, there were three contrast images generated

altogether. At the group level of analysis, we utilize these contrast images to

investigate neural activation in the reasoning timewindow by condition.

- 1. At the behavioral level, we investigated whether reasoning would be impaired by emotion manipulation. Here we show that, on average, participants did not have a significantly lower proportion of logical (correct) responses as a result of either positive or negative emotion manipulation.

Supplementary Tables

Table 1

Picture viewing / judging timewindow: Brain regions identified in the stated comparisons

| **Brain region*^a^*** | |  | | | | | | | | | | | | | | | **MNI**  **co-ordinates** | | | | | | | | | | | | | | | | | | | |  |
| --- | --- | --- | --- | --- | --- | --- | --- | --- | --- | --- | --- | --- | --- | --- | --- | --- | --- | --- | --- | --- | --- | --- | --- | --- | --- | --- | --- | --- | --- | --- | --- | --- | --- | --- | --- | --- | --- |
|  | | **k*^b^*** | | | | | ***p^c^*** | | | | | ***Z*** | | | | | **x** | | | | | | | | | | **y** | | | | | **z** | | | | |  |
| **Contrast** | | | | | | | | | | | | | | | | | | | | | | | | | | | | | | | | | | | | |  |
|  | | | | | | | | | | | | | | | | | | | | | | | | | | | | | | | | | | | | |  |
| *Emotion – Neutral* | | | | | | | | | | | | | | | | | | | | | | | | | | | | | | | | | | | | |  |
| Left middle occipital lobe (hOC5) | | 990 | | | | | .002 | | | | | 4.4 | | | | | -44 | | | | | | | | | | -70 | | | | -2 | | | | | |  |
| Left middle temporal lobe | |  | | | | |  | | | | | 3.67 | | | | | -50 | | | | | | | | | | -60 | | | | 6 | | | | | |  |
| Left fusiform gyrus | |  | | | | |  | | | | | 3.63 | | | | | -44 | | | | | | | | | | -60 | | | | -20 | | | | | |  |
| Left occipital lobe (calcarine: area 17) | | 1710 | | | | | .001 | | | | | 4.16 | | | | | 2 | | | | | | | | | | -98 | | | | 0 | | | | | |  |
| Right middle temporal lobe (hOC5) | |  | | | | |  | | | | | 4.12 | | | | | 48 | | | | | | | | | | -66 | | | | -2 | | | | | |  |
| Right inferior occipital lobe (hOC4) | |  | | | | |  | | | | | 3.47 | | | | | 36 | | | | | | | | | | -86 | | | | -6 | | | | | |  |
| Left thalamus (prefrontal) | | 284 | | | | | .157 | | | | | 3.43 | | | | | -4 | | | | | | | | | | -24 | | | | 2 | | | | | |  |
| Left pallidum | | 87 | | | | | .488 | | | | | 3.55 | | | | | -16 | | | | | | | | | | -2 | | | | 6 | | | | | |  |
| Left secondary somatosensory cortex (OP1) | | 82 | | | | | .488 | | | | | 3.66 | | | | | -50 | | | | | | | | | | -28 | | | | 18 | | | | | |  |
| Right amygdala | | 64 | | | | | .488 | | | | | 3.2 | | | | | 18 | | | | | | | | | | 0 | | | | -14 | | | | | |  |
| Left middle occipital lobe (area 18) | | 52 | | | | | .488 | | | | | 3.19 | | | | | -18 | | | | | | | | | | -102 | | | | 8 | | | | | |  |
| Right inferior parietal (supramarginal gyrus) | | 49 | | | | | .488 | | | | | 3.51 | | | | | 56 | | | | | | | | | | -22 | | | | 30 | | | | | |  |
| Right fusiform gyrus | | 37 | | | | | .514 | | | | | 3.09 | | | | | 36 | | | | | | | | | | -62 | | | | 16 | | | | | |  |
|  | |  | | | | |  | | | | |  | | | | |  | | | | | | | | | |  | | | |  | | | | | |  |
| *Neutral – Emotion* | | | | | | | | | | | | | | | | | | | | | | | | | | | | | | | | | | | | |  |
| Right inferior parietal lobe | | 733 | | | | | .062 | | | | | | 4.15 | | | | 34 | | | | | | | | | -36 | | | | | 20 | | | | |  | |
| Right superior parietal (area 7) | |  | | | | |  | | | | | | 3.7 | | | | 38 | | | | | | | | | -30 | | | | | 34 | | | | |  | |
| Right occipital lobe (lingual gyrus) | |  | | | | |  | | | | | | 3.37 | | | | 28 | | | | | | | | | -44 | | | | | -8 | | | | |  | |
| Right postcentral gyrus | | 539 | | | | | .106 | | | | | | 4.12 | | | | 36 | | | | | | | | | -34 | | | | | 70 | | | | |  | |
| Right precentral gyrus (area 6) | |  | | | | |  | | | | | | 3.74 | | | | 40 | | | | | | | | | -16 | | | | | 66 | | | | |  | |
| Right superior frontal gyrus | |  | | | | |  | | | | | | 2.96 | | | | 28 | | | | | | | | | -2 | | | | | 60 | | | | |  | |
| Left postcentral gyrus (areas 1, 3, 4) | | 132 | | | | | .917 | | | | | | 3.2 | | | | -32 | | | | | | | | | -32 | | | | | 64 | | | | |  | |
| Left parahippocampal gyrus | | 82 | | | | | .917 | | | | | | 2.87 | | | | -32 | | | | | | | | | -46 | | | | | -6 | | | | |  | |
| Left fusiform gyrus | |  | | | | |  | | | | | | 2.63 | | | | -30 | | | | | | | | | -54 | | | | | -4 | | | | |  | |
| Right middle cingulate cortex | | 59 | | | | | .917 | | | | | | 2.88 | | | | 6 | | | | | | | | | 14 | | | | | 40 | | | | |  | |
| Right superior parietal lobe (area 5) | | 49 | | | | | .917 | | | | | | 3.33 | | | | 10 | | | | | | | | | -48 | | | | | 72 | | | | |  | |
| Right frontal lobe (area 6) | | 34 | | | | | .917 | | | | | | 3.13 | | | | 8 | | | | | | | | | 4 | | | | | 66 | | | | |  | |
| Right cerebellum | | 22 | | | | | .917 | | | | | | 3.03 | | | | 8 | | | | | | | | | -52 | | | | | -18 | | | | |  | |
|  | |  | | | | |  | | | | | |  | | | |  | | | | | | | | |  | | | | |  | | | | |  | |
| *Positive – Neutral* | | | | | | | | | | | | | | | | | | | | | | | | | | | | | | | | | | | | |  |
| Left occipital lobe (hOC5) | 943 | | | | | | .005 | | | | | | 4.28 | | | | | -44 | | | | | | | | -72 | | | | | -6 | | | | |  | |
| Left fusiform gyrus |  | | | | | |  | | | | | | 3.69 | | | | | -42 | | | | | | | | -60 | | | | | -20 | | | | |  | |
| Left occipital lobe (hOC5) |  | | | | | |  | | | | | | 3.56 | | | | | -50 | | | | | | | | -62 | | | | | 6 | | | | |  | |
| Right occipital lobe (hOC5) | 1184 | | | | | | .003 | | | | | | 3.95 | | | | | 46 | | | | | | | | -68 | | | | | -4 | | | | |  | |
| Right inferior parietal lobe |  | | | | | |  | | | | | | 3.42 | | | | | 42 | | | | | | | | -58 | | | | | 14 | | | | |  | |
| SI Table 1, *continued* | | | | | | | | | | | | | | | | | | | | | | | | | | | | | | | | | | | |  | |
|  |  | | | | | | |  | | | | |  | | | | | | | |  | | | | |  | | | | |  | | | | |  | |
| *Positive – Neutral, continued* | | | | | | | | | | | | | | | | | | | | | | | | | | | | | | | | | | | |  | |
| Right cerebellum |  | | | | | |  | | | | | | 3.40 | | | | | | | | 42 | | | | | -46 | | | | | -30 | | | | |  | |
| Left occipital lobe (calcarine: area 17) | 189 | | | | | | .412 | | | | | | 3.44 | | | | | | | | -2 | | | | | -94 | | | | | 2 | | | | |  | |
| Left occipital lobe (calcarine: area 17) | |  | | | | |  | | | | | | 3.21 | | | | | | | | -8 | | | | | -90 | | | | | -2 | | | | |  | |
| Left parietal (supramarginal gyrus and  secondary somatosensory area) | | 117 | | | | | .589 | | | | | | 4.52 | | | | | | | | -50 | | | | | -28 | | | | | 20 | | | | |  | |
| Right cerebellum | | 60 | | | | | .923 | | | | | | 4.12 | | | | | | | | 20 | | | | | -78 | | | | | -22 | | | | |  | |
| Left thalamus | | 26 | | | | | .923 | | | | | | 3.0 | | | | | | | | -14 | | | | | -28 | | | | | -6 | | | | |  | |
|  | |  | | | | |  | | | | | |  | | | | | | | |  | | | | |  | | | | |  | | | | |  | |
| *Neutral – Positive* | | | | | | | | | | | | | | | | | | | | | | | | | | | | | | | | | | | | |  |
| Right middle frontal gyrus | | 2773 | | | | | .001 | | | | | | 4.3 | | | | | | | | 26 | | | | | 28 | | | | | 32 | | | | |  | |
| Left middle frontal gyrus | |  | | | | |  | | | | | | 4.25 | | | | | | | | -26 | | | | | 34 | | | | | 32 | | | | |  | |
| Right frontal (area 6) | | 1037 | | | | | .004 | | | | | | 3.87 | | | | | | | | 30 | | | | | -18 | | | | | 66 | | | | |  | |
| Right frontal (area 4) | |  | | | | |  | | | | | | 3.79 | | | | | | | | 24 | | | | | -34 | | | | | 70 | | | | |  | |
| Right frontal (area 4) | |  | | | | |  | | | | | | 3.44 | | | | | | | | 6 | | | | | -30 | | | | | 70 | | | | |  | |
| Right parahippocampal gyrus | | 1080 | | | | | .004 | | | | | | 3.61 | | | | | | | | 30 | | | | | -42 | | | | | -4 | | | | |  | |
| Left inferior parietal (supramarginal gyrus, angular gyrus) | | 166 | | | | | .593 | | | | | | 3.72 | | | | | | | | -54 | | | | | -56 | | | | | 40 | | | | |  | |
| Left hippocampus (CA) | | 124 | | | | | .780 | | | | | | 3.59 | | | | | | | | -32 | | | | | -36 | | | | | -6 | | | | |  | |
| Left hippocampus (CA) | |  | | | | |  | | | | | | 3.27 | | | | | | | | -32 | | | | | -32 | | | | | -16 | | | | |  | |
| Right precuneus | | 72 | | | | | .877 | | | | | | 2.68 | | | | | | | | 16 | | | | | -60 | | | | | 42 | | | | |  | |
| Right frontal lobe (area 6) | | 65 | | | | | .877 | | | | | | 3.03 | | | | | | | | 8 | | | | | 4 | | | | | 66 | | | | |  | |
| Right frontal lobe (areas 6, 4) | | 64 | | | | | .877 | | | | | | 3.04 | | | | | | | | 54 | | | | | -8 | | | | | 42 | | | | |  | |
| Right frontal lobe (area 6) | |  | | | | |  | | | | | | 2.96 | | | | | | | | 46 | | | | | -6 | | | | | 48 | | | | |  | |
| Right postcentral gyrus (area 2) | | 50 | | | | | .917 | | | | | | 3.17 | | | | | | | | 34 | | | | | -48 | | | | | 64 | | | | |  | |
| Right postcentral gyrus (area 1) | |  | | | | |  | | | | | | 2.98 | | | | | | | | 40 | | | | | -42 | | | | | 62 | | | | |  | |
| Left precuneus | | 44 | | | | | .917 | | | | | | 3.15 | | | | | | | | -10 | | | | | -54 | | | | | 50 | | | | |  | |
| Right cerebellum | | 31 | | | | | .917 | | | | | | 3.02 | | | | | | | | 10 | | | | | -48 | | | | | -20 | | | | |  | |
| Left insula lobe | | 27 | | | | | .917 | | | | | | 2.81 | | | | | | | | -38 | | | | | 12 | | | | | 4 | | | | |  | |
| Left frontal lobe (area 6) | | 26 | | | | | .917 | | | | | | 2.87 | | | | | | | | -10 | | | | | -22 | | | | | 64 | | | | |  | |
|  | |  | | | | |  | | | | | |  | | | | | | | |  | | | | |  | | | | |  | | | | |  | |
| *Negative – Neutral* | | | | | | | | | | | | | | | | | | | | | | | | | | | | | | | | | | | | |  |
| Left occipital lobe (calcarine: area 17) | | 1128 | | | | | .002 | | | | | | 4.29 | | | | | | | | 0 | | | | | -98 | | | | | 2 | | | | |  | |
| Left occipital lobe (lingual: area 18) | |  | | | | |  | | | | | | 3.94 | | | | | | | | -10 | | | | | -88 | | | | | -12 | | | | |  | |
| Right mid-occipital lobe (hOC4v) | |  | | | | |  | | | | | | 3.37 | | | | | | | | 34 | | | | | -92 | | | | | 0 | | | | |  | |
| Left middle occipital gyrus | | 541 | | | | | .033 | | | | | | 3.69 | | | | | | | | -44 | | | | | -68 | | | | | 0 | | | | |  | |
| Left fusiform gyrus | |  | | | | |  | | | | | | 3.09 | | | | | | | | -42 | | | | | -58 | | | | | -20 | | | | |  | |
| Left inferior occipital (hOC4v) | |  | | | | |  | | | | | | 2.74 | | | | | | | | -40 | | | | | -78 | | | | | -18 | | | | |  | |
| Left putamen | | 155 | | | | | .262 | | | | | | 4.54 | | | | | | | | -18 | | | | | 0 | | | | | 10 | | | | |  | |
| Right amygdala | | 126 | | | | | .282 | | | | | | 3.77 | | | | | | | | 18 | | | | | 2 | | | | | -14 | | | | |  | |
| Right inferior parietal (supramarginal gyrus) | | 122 | | | | | .282 | | | | | | 4.14 | | | | | | | | 56 | | | | | -20 | | | | | 30 | | | | |  | |
| Right inferior occipital (hOC: V5) | | 102 | | | | | .316 | | | | | | 3.32 | | | | | | | | 48 | | | | | -64 | | | | | 2 | | | | |  | |
| Right inferior frontal gyrus (triangularis, area 45) | | 45 | | | | | .618 | | | | | | 3.47 | | | | | | | | 48 | | | | | 28 | | | | | 8 | | | | |  | |
| Left inferior parietal (secondary somatosensory cortex, supramarginal gyrus) | | 20 | | | | | .828 | | | | | | 2.78 | | | | | | | | -52 | | | | | -28 | | | | | 18 | | | | |  | |
| SI Table 1, *continued* | | | | | | | | | | | | | | | | | | | | | | | | | | | | | | | | | | | | |  |
|  | | | | | | | | | | | | | | | | | | | | | | | | | | | | | | | | | | | | |  |
| *Neutral - Negative* | | | | | | | | | | | | | | | | | | | | | | | | | | | | | | | | | | | | |  |
| Left postcentral gyrus (area 1) | | | 150 | | | | .805 | | | | | | 3.15 | | | | | | -30 | | | | | | -36 | | | | | | | 68 | | | | |  |
| Left postcentral gyrus (area 1) | | |  | | | |  | | | | | | 3.01 | | | | | | -40 | | | | | | -34 | | | | | | | 66 | | | | |  |
| Right middle cingulate cortex | | | 62 | | | | .805 | | | | | | 3.31 | | | | | | 6 | | | | | | 18 | | | | | | | 40 | | | | |  |
| Right precentral gyrus (area 6) | | | 49 | | | | .805 | | | | | | 3.00 | | | | | | 40 | | | | | | -12 | | | | | | | 64 | | | | |  |
| Right inferior occipital (calcarine: area 17) | | | 47 | | | | .805 | | | | | | 2.99 | | | | | | 22 | | | | | | -60 | | | | | | | 10 | | | | |  |
| Left postcentral gyrus (areas 1, 4) | | | 32 | | | | .805 | | | | | | 3.34 | | | | | | -52 | | | | | | -14 | | | | | | | 48 | | | | |  |
|  | | |  | | | |  | | | | | |  | | | | | |  | | | | | |  | | | | | | |  | | | | |  |
| *Positive - Negative* | | | | | | | | | | | | | | | | | | | | | | | | | | | | | | | | | | | | |  |
| Left superior temporal gyrus | | | 103 | | | | .922 | | | | | | 3.04 | | | | | | | -66 | | | | | -40 | | | | | | | 16 | | | | |  |
| Left superior temporal gyrus | | |  | | | |  | | | | | | 3.03 | | | | | | | -60 | | | | | -44 | | | | | | | 12 | | | | |  |
| Right superior temporal gyrus | | | 61 | | | | .922 | | | | | | 3.12 | | | | | | | 60 | | | | | -42 | | | | | | | 12 | | | | |  |
| Right hippocampus (EC) | | | 38 | | | | .922 | | | | | | 3.05 | | | | | | | 26 | | | | | 4 | | | | | | | -38 | | | | |  |
| Left cerebellum | | | 32 | | | | .922 | | | | | | 2.92 | | | | | | | -4 | | | | | -44 | | | | | | | -22 | | | | |  |
| Left postcentral gyrus (areas 1, 2) | | | 26 | | | | .922 | | | | | | 3.43 | | | | | | | -38 | | | | | -42 | | | | | | | 66 | | | | |  |
|  | | |  | | | |  | | | | | |  | | | | | | |  | | | | |  | | | | | | |  | | | | |  |
| *Negative – Positive* | | | | | | | | | | | | | | | | | | | | | | | | | | | | | | | | | | | | |  |
| Left amygdala | | | | 552 | | | .082 | | | | 3.74 | | | | | | | | | -28 | | | | | | -2 | | | | | | | -10 | | | | |
| Left insula lobe | | | |  | | |  | | | | 3.38 | | | | | | | | | -28 | | | | | | 18 | | | | | | | 8 | | | | |
| Left insula lobe | | | |  | | |  | | | | 2.7 | | | | | | | | | -36 | | | | | | 6 | | | | | | | 0 | | | | |
| Right precuneus | | | | 404 | | | .147 | | | | 3.09 | | | | | | | | | 16 | | | | | | -44 | | | | | | | 12 | | | | |
| Right precuneus | | | |  | | |  | | | | 2.9 | | | | | | | | | 20 | | | | | | -50 | | | | | | | 22 | | | | |
| Right precentral gyrus (area 44) | | | | 370 | | | .147 | | | | 3.39 | | | | | | | | | 42 | | | | | | 4 | | | | | | | 38 | | | | |
| Right inferior frontal gyrus (area 44) | | | |  | | |  | | | | 3.13 | | | | | | | | | 36 | | | | | | 10 | | | | | | | 28 | | | | |
| Right superior frontal gyrus | | | |  | | |  | | | | 3.07 | | | | | | | | | 18 | | | | | | 30 | | | | | | | 34 | | | | |
| Left middle cingulate cortex | | | | 166 | | | .515 | | | | 3.07 | | | | | | | | | -10 | | | | | | -28 | | | | | | | 34 | | | | |
| Right inferior parietal (supramarginal gyrus) | | | | 161 | | | .515 | | | | 3.66 | | | | | | | | | 48 | | | | | | -22 | | | | | | | 30 | | | | |
| Right postcentral gyrus (area 2) | | | |  | | |  | | | | 2.98 | | | | | | | | | 46 | | | | | | -22 | | | | | | | 42 | | | | |
| Right postcentral gyrus (area 1) | | | |  | | |  | | | | 2.75 | | | | | | | | | 48 | | | | | | -20 | | | | | | | 50 | | | | |
| Left middle occipital gyrus | | | | 157 | | | .515 | | | | 3.26 | | | | | | | | | -22 | | | | | | -94 | | | | | | | 0 | | | | |
| Left inferior occipital (hOC3v) | | | |  | | |  | | | | 3.19 | | | | | | | | | -18 | | | | | | -82 | | | | | | | -10 | | | | |
| Left inferior occipital lobe (area 18) | | | |  | | |  | | | | 2.99 | | | | | | | | | -8 | | | | | | -90 | | | | | | | -6 | | | | |
| Left supramarginal gyrus | | | | 126 | | | .609 | | | | 3.18 | | | | | | | | | -56 | | | | | | -24 | | | | | | | -30 | | | | |
| Left superior frontal gyrus | | | | 117 | | | .609 | | | | 3.55 | | | | | | | | | -16 | | | | | | 10 | | | | | | | 56 | | | | |
| Left frontal (supplementary motor area) | | | |  | | |  | | | | 2.98 | | | | | | | | | -8 | | | | | | 18 | | | | | | | 60 | | | | |
| Left inferior parietal (supramarginal gyrus) | | | | 104 | | |  | | | | 3.64 | | | | | | | | | -42 | | | | | | -54 | | | | | | | 36 | | | | |
| Left inferior occipital (lingual gyrus) | | | | 74 | | |  | | | | 3.2 | | | | | | | | | -10 | | | | | | -32 | | | | | | | -4 | | | | |
| Left superior parietal (area 7) | | | | 57 | | |  | | | | 3.1 | | | | | | | | | 0 | | | | | | -62 | | | | | | | 52 | | | | |
| Right postcentral gyrus (areas 3, 4) | | | | 41 | | |  | | | | 2.9 | | | | | | | | | 22 | | | | | | -36 | | | | | | | 68 | | | | |
| Right hippocampus (CA) | | | | 38 | | |  | | | | 3.07 | | | | | | | | | 30 | | | | | | -40 | | | | | | | 0 | | | | |
| Left frontal lobe (area 4) | | | | 29 | | |  | | | | 3.08 | | | | | | | | | -4 | | | | | | -36 | | | | | | | -60 | | | | |
| Right frontal (precentral gyrus: area 6) | | | | 28 | | |  | | | | 2.97 | | | | | | | | | 24 | | | | | | -20 | | | | | | | 62 | | | | |
| Left inferior frontal gyrus (opercularis: area 44) | | | | 27 | | |  | | | | 2.99 | | | | | | | | | -48 | | | | | | 8 | | | | | | | 24 | | | | |
|  | | | |  | | |  | | | |  | | | | | | | | |  | | | | | |  | | | | | | |  | | | | |
| SI Table 1, *continued* | | | | | | | | | | | | | | | | | | | | | | | | | | | | | | | | | | | | |  |
|  | | | | | |  | | |  | | | | |  | | | | | | | |  | | | | | | | |  | | |  | | | | |
| *Parametric analysis: Correlations with increasingly positive intensity ratings* | | | | | | | | | | | | | | | | | | | | | | | | | | | | | | | | | | | | |  |
| Right inferior temporal gyrus | | | | | 1246 | | | | | .002 | | | | | 5.36 | | | | | | | | 48 | | | | | | -74 | | | | | | -10 | | |
| Right middle temporal gyrus | | | | |  | | | | |  | | | | | 5.35 | | | | | | | | 58 | | | | | | -68 | | | | | | -2 | | |
| Right fusiform gyrus | | | | |  | | | | |  | | | | | 4.42 | | | | | | | | 42 | | | | | | -56 | | | | | | -20 | | |
| Left occipital (calcarine: area 17) | | | | | 1128 | | | | | .002 | | | | | 4.65 | | | | | | | | -4 | | | | | | -100 | | | | | | 8 | | |
| Right occipital (area 18) | | | | |  | | | | |  | | | | | 3.93 | | | | | | | | 28 | | | | | | -102 | | | | | | 8 | | |
| Right cerebellum | | | | |  | | | | |  | | | | | 3.79 | | | | | | | | 2 | | | | | | -58 | | | | | | -2 | | |
| Left inferior occipital (hOC4) | | | | | 375 | | | | | .07 | | | | | 3.85 | | | | | | | | -36 | | | | | | -84 | | | | | | -10 | | |
| Left inferior occipital | | | | |  | | | | |  | | | | | 3.84 | | | | | | | | -46 | | | | | | -80 | | | | | | -10 | | |
| Left middle temporal gyrus | | | | |  | | | | |  | | | | | 3.47 | | | | | | | | -54 | | | | | | -68 | | | | | | 4 | | |
| Left inferior frontal (orbitalis) | | | | | 310 | | | | | .089 | | | | | 3.54 | | | | | | | | -36 | | | | | | 24 | | | | | | -8 | | |
| Left inferior frontal (areas 44, 45) | | | | |  | | | | |  | | | | | 2.97 | | | | | | | | -48 | | | | | | 20 | | | | | | 0 | | |
| Left thalamus (temporal, prefrontal) | | | | | 221 | | | | | .146 | | | | | 4.45 | | | | | | | | -10 | | | | | | -4 | | | | | | -12 | | |
| Left postcentral gyrus | | | | | 157 | | | | | .217 | | | | | 3.29 | | | | | | | | -44 | | | | | | -28 | | | | | | 66 | | |
| Left cerebellum | | | | | 139 | | | | | .225 | | | | | 3.97 | | | | | | | | -34 | | | | | | -62 | | | | | | -20 | | |
| Left cerebellum | | | | |  | | | | |  | | | | | 3.35 | | | | | | | | -34 | | | | | | -54 | | | | | | -24 | | |
| Left cerebellum | | | | |  | | | | |  | | | | | 3.15 | | | | | | | | -26 | | | | | | -56 | | | | | | -22 | | |
| Right postcentral gyrus (area 1) | | | | | 53 | | | | | .459 | | | | | 2.97 | | | | | | | | 46 | | | | | | -26 | | | | | | 60 | | |
| Right hippocampus (EC) | | | | | 53 | | | | | .459 | | | | | 3.22 | | | | | | | | 26 | | | | | | 2 | | | | | | -36 | | |
|  | | | | |  | | | | |  | | | | |  | | | | | | | |  | | | | | |  | | | | | |  | | |
| *Parametric analysis: Correlations with increasingly negative intensity ratings* | | | | | | | | | | | | | | | | | | | | | | | | | | | | | | | | | | | | |  |
| Right occipital (lingual gyrus, area 18) | | | | | 11243 | | | .001 | | | | | | | | 6.33 | | | | | | | | 10 | | | | -86 | | | | | | -6 | | |  |
| Right occipital (calcarine: area 17) | | | | |  | | |  | | | | | | | | 5.84 | | | | | | | | 12 | | | | -98 | | | | | | 4 | | |  |
| Right occipital (cuneus: areas 17, 18) | | | | |  | | |  | | | | | | | | 5.28 | | | | | | | | 22 | | | | -94 | | | | | | 10 | | |  |
| Right amygdala | | | | | 744 | | | .009 | | | | | | | | 3.81 | | | | | | | | 20 | | | | -6 | | | | | | -16 | | |  |
| Right inferior frontal gyrus (triangularis: area 45) | | | | | 57 | | | .894 | | | | | | | | 3.31 | | | | | | | | 52 | | | | 32 | | | | | | 10 | | |  |

*^a^*Brain regions have been identified using SPM Anatomy Toolbox (Eickhoff et al., 2005)

*^b^*All reported regions are significant at p < .005 for 20 contiguous voxels uncorrected.

*^c^*At the Associate Editor’s request, we also provide the cluster *p*-value, corrected for multiple comparisons using the false discovery rate (FDR; Genovese, Lazar, & Nichols, 2002).

Areas of peak activity include cluster size and are followed by other significant areas of activation in that cluster.

For Table 2, see next page .....

Table 2

Reasoning timewindow: Brain regions identified in the stated comparisons

| **Brain region*^a^*** |  | | | | | | | **MNI**  **co-ordinates** | | | | | | | | | |  | | | |
| --- | --- | --- | --- | --- | --- | --- | --- | --- | --- | --- | --- | --- | --- | --- | --- | --- | --- | --- | --- | --- | --- |
|  | **k*^b^*** | ***p^c^*** | ***Z*** | | | | | **x** | | | **y** | | | **z** | | | |  | | | |
| **Contrast** | | | | | | | | | | | | | | | | | |  | | | |
| *Reasoning – Baseline* | | | | | | | | | | | | | | | | | |  | | | |
| Right occipital lobe (calcarine gyrus, area 17) | 693 | .043 | | | 5.56 | | | 20 | | | -96 | | | 0 | | | |  | | | |
| Right inferior occipital (Hoc3v, Hoc4v) |  |  | | | 4.44 | | | 34 | | | -88 | | | -4 | | | |  | | | |
| Right lingual gyrus (Hoc3v) |  |  | | | 3.56 | | | 24 | | | -80 | | | -8 | | | |  | | | |
| Left occipital lobe (calcarine gyrus, area 18) | 538 | .052 | | | 4.14 | | | -8 | | | -92 | | | -4 | | | |  | | | |
| Left inferior occipital (Hoc4v) |  |  | | | 3.56 | | | -32 | | | -84 | | | -6 | | | |  | | | |
| Right thalamus (prefrontal) | 503 | .052 | | | 3.48 | | | 4 | | | -20 | | | 0 | | | |  | | | |
| Left postcentral gyrus (areas 4, 3) | 42 | .700 | | | 3.06 | | | -38 | | | -18 | | | 44 | | | |  | | | |
|  | | | | | | | | | | | | | | | | | |  | | | |
| *Emotional Reasoning – Emotional Baseline* | | | | | | | | | | | | | | | | | |  | | | |
| Right occipital (calcarine: area 17) | 635 | .204 | | | 5.92 | | | 20 | | | -96 | | | 0 | | | |  | | | |
| Right inferior occipital gyrus (Hoc3v. Hoc4v) |  |  | | | 4.33 | | | 30 | | | -84 | | | -6 | | | |  | | | |
| Left occipital (calcarine: area 17) | 396 | .21 | | | 3.6 | | | -10 | | | -96 | | | -2 | | | |  | | | |
| Left postcentral gyrus (area 4) | 187 | .568 | | | 3.66 | | | -38 | | | -20 | | | 46 | | | |  | | | |
| Left inferior occipital gyrus (Hoc4v) | 94 | .901 | | | 3.38 | | | -32 | | | -86 | | | -6 | | | |  | | | |
| Left middle temporal gyrus | 91 | .901 | | | 3.26 | | | -58 | | | -36 | | | 4 | | | |  | | | |
| Left supramarginal gyrus | 37 | .903 | | | 3.34 | | | -66 | | | -22 | | | 34 | | | |  | | | |
| Left middle temporal gyrus | 28 | .903 | | | 2.78 | | | -48 | | | -60 | | | 18 | | | |  | | | |
|  | | | | | | | | | | | | | | | | | |  | | | |
| *Positive Reasoning – Positive Baseline* | | | | | | | | | | | | | | | | | |  | | | |
| Right occipital (calcarine: area 17) | 665 | .138 | | | 4.62 | | | 20 | | | -96 | | | 0 | | | |  | | | |
| Right lingual (area 17) |  |  | | | 4.16 | | | 16 | | | -90 | | | -4 | | | |  | | | |
| Right inferior occipital gyrus (Hoc4v. Hoc3v) |  |  | | | 3.79 | | | 28 | | | -84 | | | -6 | | | |  | | | |
| Right thalamus (prefrontal) | 114 | .938 | | | 3.01 | | | 6 | | | -22 | | | -4 | | | |  | | | |
| Right middle temporal gyrus | 62 | .938 | | | 3.24 | | | 48 | | | 0 | | | -16 | | | |  | | | |
| Right precentral gyrus (areas 4, 6) | 42 | .938 | | | 2.98 | | | 26 | | | -30 | | | 70 | | | |  | | | |
| Left supramarginal gyrus | 36 | .938 | | | 3.29 | | | -64 | | | -26 | | | 40 | | | |  | | | |
|  |  |  | | |  | | |  | | |  | | |  | | | |  | | | |
| *Negative Reasoning – Negative Baseline* | | | | | | | | | | | | | | | | | |  | | | |
| Right occipital lobe (calcarine: area 17) | 383 | .72 | | | 5.19 | | | 20 | | | -96 | | | 2 | | | |  | | | |
| Right inferior occipital gyrus |  |  | | | 3.82 | | | 38 | | | -88 | | | -4 | | | |  | | | |
| Right inferior occipital gyrus |  |  | | | 2.91 | | | 44 | | | -82 | | | -6 | | | |  | | | |
| Left postcentral gyrus (area 4) | 230 | .894 | | | 4.27 | | | -38 | | | -20 | | | 44 | | | |  | | | |
| Left occipital lobe (calcarine: area 17) | 86 | .894 | | | 3.04 | | | -8 | | | -92 | | | 2 | | | |  | | | |
| Left inferior frontal gyrus (triangularis) | 61 | .894 | | | 2.98 | | | -38 | | | 14 | | | 30 | | | |  | | | |
| Left inferior occipital gyrus (hOC4v) | 44 | .894 | | | 3.0 | | | -34 | | | -84 | | | -6 | | | |  | | | |
| Left supramarginal gyrus | 39 | .894 | | | 2.75 | | | -58 | | | -18 | | | 30 | | | |  | | | |
| Left middle temporal gyrus | 20 | .894 | | | 2.75 | | | -50 | | | -54 | | | 16 | | | |  | | | |
|  |  |  | | |  | | |  | | |  | | |  | | | |  | | | |
| SI Table 2 *continued* | | | | | | | | | | | | | | | | | |  | | | |
|  |  |  | | |  | | |  | | |  | | |  | | | |  | | | |
| *Neutral Reasoning – Neutral Baseline* | | | | | | | | | | | | | | | | | |  | | | |
| Left middle temporal gyrus | 105 | .743 | | | 3.41 | | | -62 | | | -54 | | | 12 | | | |  | | | |
| Left occipital lobe (calcarine: area 18) | 72 | .743 | | | 3.15 | | | -8 | | | -92 | | | -4 | | | |  | | | |
| Right occipital lobe (calcarine: area 17) | 34 | .743 | | | 3.06 | | | 16 | | | -102 | | | 2 | | | |  | | | |
| Right thalamus (prefrontal) | 31 | .743 | | | 2.8 | | | 6 | | | -14 | | | 2 | | | |  | | | |
|  |  |  | | |  | | |  | | |  | | |  | | | |  | | | |
| *Emotional Reasoning – Neutral Reasoning* | | | | | | | | | | | | | | | | | |  | | | |
| Left hippocampus (EC) | 165 | .903 | | | 3.11 | | | -28 | | | -16 | | | -34 | | | |  | | | |
| Left inferior temporal gyrus |  |  | | | 3.04 | | | -46 | | | -20 | | | -32 | | | |  | | | |
| Left inferior temporal gyrus |  |  | | | 3.01 | | | -46 | | | -28 | | | -26 | | | |  | | | |
| Right supramarginal gyrus | 91 | .903 | | | 3.61 | | | 56 | | | -34 | | | 54 | | | |  | | | |
| Right occipital lobe (calcarine: area 17) | 55 | .903 | | | 2.87 | | | 16 | | | -90 | | | 0 | | | |  | | | |
|  |  |  | | |  | | |  | | |  | | |  | | | |  | | | |
| *Neutral Reasoning – Emotional Reasoning* | | | | | | | | | | | | | | | | | |  | | | |
| Left insula lobe | 298 | .663 | | | 3.19 | | | -42 | | | -8 | | | 6 | | | |  | | | |
| Left insula |  |  | | | 3.17 | | | -34 | | | -24 | | | 8 | | | |  | | | |
| Right pallidum | 122 | .887 | | | 3.05 | | | 22 | | | 0 | | | 4 | | | |  | | | |
| Right putamen |  |  | | | 2.76 | | | 30 | | | 4 | | | 8 | | | |  | | | |
| Right inferior frontal gyrus (triangularis) | 114 | .887 | | | 3.65 | | | 46 | | | 36 | | | 6 | | | |  | | | |
| Left precentral gyrus | 32 | .887 | | | 2.98 | | | -46 | | | -4 | | | 28 | | | |  | | | |
| Left superior temporal gyrus (TE 3) | 32 | .887 | | | 2.82 | | | -62 | | | -18 | | | 2 | | | |  | | | |
| Right frontal lobe (area 6) | 26 | .887 | | | 3.07 | | | 24 | | | -20 | | | 48 | | | |  | | | |
| Right thalamus (prefrontal) | 25 | .887 | | | 2.9 | | | 6 | | | -10 | | | 2 | | | |  | | | |
|  |  |  | | |  | | |  | | |  | | |  | | | |  | | | |
| *Positive Reasoning – Neutral Reasoning* | | | | | | | | | | | | | | | | | |  | | | |
| Right inferior parietal (supramarginal gyrus) | 53 | .715 | | | 3.19 | | | 54 | | | -34 | | | 54 | | | |  | | | |
|  |  |  | | |  | | |  | | |  | | |  | | | |  | | | |
| *Neutral Reasoning – Positive Reasoning* | | | | | | | | | | | | | | | | | |  | | | |
| Left inferior frontal gyrus (orbitalis) | 217 | .637 | | | 3.73 | | | -42 | | | 20 | | | -4 | | | |  | | | |
| Right inferior frontal gyrus (triangularis) | 201 | .637 | | | 4.06 | | | 48 | | | 36 | | | 6 | | | |  | | | |
| Right occipital lobe (calcarine) | 164 | .637 | | | 3.19 | | | 30 | | | -56 | | | 12 | | | |  | | | |
| Left insula lobe | 106 | .776 | | | 3.03 | | | -32 | | | -22 | | | 8 | | | |  | | | |
| Left precentral gyrus | 73 | .938 | | | 3.28 | | | -44 | | | -4 | | | 28 | | | |  | | | |
| Right paracentral lobule (areas 4, superior parietal 5) | 45 | .938 | | | 3.05 | | | 2 | | | -38 | | | 66 | | | |  | | | |
| Left superior temporal gyrus (TE 3) | 43 | .938 | | | 2.92 | | | -64 | | | -18 | | | 2 | | | |  | | | |
| Right frontal lobe (area 6) | 42 | .938 | | | 3.23 | | | 24 | | | -20 | | | 48 | | | |  | | | |
|  |  |  | | |  | | |  | | |  | | |  | | | |  | | | |
| *Negative Reasoning - Neutral Reasoning* | | | | | | | | | | | | | | | | | |  | | | |
| Right occipital lobe (calcarine: area 17) | 146 | .779 | | | 3.5 | | | 18 | | | -92 | | | 0 | | | |  | | | |
| Left middle frontal gyrus | 98 | .779 | | | 3.16 | | | -46 | | | 46 | | | 16 | | | |  | | | |
| Left middle frontal gyrus |  |  | | | 2.99 | | | -44 | | | 54 | | | 6 | | | |  | | | |
| Left hippocampus (EC) | 81 | .779 | | | 3.23 | | | -26 | | | -16 | | | -36 | | | |  | | | |
| Left inferior temporal gyrus |  |  | | | 2.71 | | | -40 | | | -14 | | | -38 | | | |  | | | |
| Left postcentral gyrus (areas 4, 3) | 78 | .779 | | | 3.19 | | | -36 | | | -22 | | | 48 | | | |  | | | |
| SI Table 2 *continued* | | | | | | | | | | | | | | | | | |  | | | |
|  |  |  | | |  | | |  | | |  | | |  | | | |  | | | |
| *Negative Reasoning - Neutral Reasoning, continued* | | | | | | | | | | | | | | | | | |  | | | |
| Right frontal (area 6) | 74 | .779 | | | 3.12 | | | 8 | | | -24 | | | 48 | | | |  | | | |
| Right parietal (supramarginal gyrus) | 65 | .779 | | | 3.41 | | | 56 | | | -34 | | | 54 | | | |  | | | |
| Right middle temporal gyrus | 51 | .779 | | | 3.0 | | | 46 | | | -66 | | | 16 | | | |  | | | |
| Left occipital lobe (calcarine) | 27 | .779 | | | 2.98 | | | -6 | | | -60 | | | 8 | | | |  | | | |
| Left superior parietal lobe (area 7) | 25 | .779 | | | 2.92 | | | -38 | | | -48 | | | 56 | | | |  | | | |
|  |  |  | | |  | | |  | | |  | | |  | | | |  | | | |
| *Neutral Reasoning – Negative Reasoning* | | | | | | | | | | | | | | | | | |  | | | |
| Left insula lobe | 375 | .395 | | | 4.14 | | | -42 | | | -8 | | | | 6 | | | |  | | |
| Left Heschls gyrus (TE 1.1) |  |  | | | 2.72 | | | -36 | | | -24 | | | | 8 | | | |  | | |
| Right thalamus (prefrontal) | 253 | .426 | | | 3.82 | | | 6 | | | -8 | | | | 0 | | | |  | | |
| Right pallidum |  |  | | | 3.21 | | | 22 | | | 0 | | | | 4 | | | |  | | |
| Right superior temporal gyrus | 36 | .847 | | | 3.02 | | | 44 | | | 0 | | | | -14 | | | |  | | |
|  |  |  | | |  | | |  | | |  | | | |  | | | |  | | |
| *Positive Reasoning – Negative Reasoning* | | | | | | | | | | | | | | | | | |  | | | |
| Right inferior frontal gyrus (orbitalis) | 234 | .557 | | | 3.73 | | | 44 | | | 40 | | | | -16 | | | |  | | |
| Right inferior frontal gyrus (orbitalis) |  |  | | | 3.07 | | | 56 | | | 32 | | | | -10 | | | |  | | |
| Right superior temporal gyrus | 144 | .557 | | | 4.08 | | | 44 | | | -2 | | | | -14 | | | |  | | |
| Left insula lobe | 72 | .557 | | | 3.16 | | | -38 | | | -6 | | | | 6 | | | |  | | |
| Left superior temporal gyrus | 67 | .557 | | | 3.03 | | | -44 | | | -4 | | | | -8 | | | |  | | |
| Right thalamus (temporal, prefrontal) | 38 | .647 | | | 3.08 | | | 4 | | | -8 | | | | 0 | | | |  | | |
|  |  |  | | |  | | |  | | |  | | | |  | | | |  | | |
| *Negative Reasoning – Positive Reasoning* | | | | | | | | | | | | | | | | | | | | |  |
| Left caudate nucleus | 1294 | .017 | | 4.3 | | | -12 | | | 2 | | | | | | | 20 | | |  | |
| Left insula lobe |  |  | | 3.62 | | | -26 | | | 30 | | | | | | | 12 | | |  | |
| Right caudate nucleus | 300 | .781 | | 3.48 | | | 20 | | | 2 | | | | | | | 20 | | |  | |
| Right occipital lobe (cuneus: areas 18, 17) | 232 | .781 | | 3.6 | | | 14 | | | -100 | | | | | | | 14 | | |  | |
| Right occipital lobe (calcarine: area 17) |  |  | | 2.76 | | | 20 | | | -92 | | | | | | | 2 | | |  | |
| Left superior occipital gyrus | 228 | .781 | | 3.51 | | | -16 | | | -96 | | | | | | | 26 | | |  | |
| Left superior occipital gyrus (area 17) |  |  | | 2.84 | | | -6 | | | -102 | | | | | | | 12 | | |  | |
| Left postcentral gyrus (area 4) | 149 | .923 | | 3.32 | | | -34 | | | -20 | | | | | | | 44 | | |  | |
| Left precuneus | 106 | .923 | | 3.25 | | | -10 | | | -52 | | | | | | | 10 | | |  | |
| Left occipital lobe (lingual gyrus) | 48 | .938 | | 2.91 | | | -26 | | | -66 | | | | | | | -8 | | |  | |
| Right superior occipital gyrus | 36 | .938 | | 3.07 | | | 30 | | | -80 | | | | | | | 38 | | |  | |
|  |  |  | |  | | |  | | |  | | | | | | |  | | |  | |
| *(Emotional reasoning – emotional baseline) –*  *(Neutral reasoning – neutral baseline)* | | | | | | | | | | | | | | | | | | | | |  |
| Left postcentral gyrus (areas 3, 4) | 854 | .073 | | 3.89 | | | -32 | | | -34 | | | 58 | | | | | | |  | |
| Left postcentral gyrus (area 2) |  |  | | 3.32 | | | -44 | | | -32 | | | 48 | | | | | | |  | |
| Right supplementary motor (area 6) | 579 | .135 | | 4.44 | | | 6 | | | -22 | | | 50 | | | | | | |  | |
| Left superior parietal lobe (area 5) |  |  | | 3.78 | | | -8 | | | -30 | | | 50 | | | | | | |  | |
| Left inferior parietal (angular gyrus) | 245 | .59 | | 3.63 | | | -46 | | | -68 | | | 26 | | | | | | |  | |
| Left middle occipital gyrus |  |  | | 3.12 | | | -34 | | | -80 | | | 30 | | | | | | |  | |
| Left inferior frontal gyrus (triangularis) | 182 | .59 | | 3.4 | | | -42 | | | 24 | | | 26 | | | | | | |  | |
| SI Table 2 *continued* | | | | | | | | | | | | | | | | | | | |  | |
|  |  |  | |  | | |  | | |  | | |  | | | | | | |  | |
| *(Emotional reasoning – emotional baseline) –*  *(Neutral reasoning – neutral baseline), continued* | | | | | | | | | | | | | | | | | | | |  | |
| Right parietal lobe (angular gyrus) | 61 | .918 | | 3.07 | | | 48 | | | -64 | | | 20 | | | | | | |  | |
| Right occipital lobe (calcarine: area 17) | 32 | .918 | | 2.9 | | | 18 | | | -90 | | | 0 | | | | | | |  | |
| Right occipital lobe (cuneus) | 23 | .918 | | 2.82 | | | 12 | | | -72 | | | 24 | | | | | | |  | |
| Right parietal lobe (angular gyrus) | 21 | .918 | | 2.86 | | | 44 | | | -80 | | | 32 | | | | | | |  | |
|  |  |  | |  | | |  | | |  | | |  | | | | | | |  | |
| *(Neutral reasoning – neutral baseline) –*  *(Emotional reasoning – emotional baseline)* | | | | | | | | | | | | | | | | | | | | |  |
| Right hippocampus (EC) | 62 | .795 | | 2.95 | | | 20 | | | -4 | | | -28 | | | | | | |  | |
| Left middle temporal gyrus | 43 | .795 | | 2.97 | | | -62 | | | -56 | | | 10 | | | | | | |  | |
|  |  |  | |  | | |  | | |  | | |  | | | | | | |  | |
| *(Positive reasoning – positive baseline) –*  *(Neutral reasoning – neutral baseline)* | | | | | | | | | | | | | | | | | | | | |  |
| Right supplementary motor (area 6) | 317 | .654 | | 3.34 | | | 6 | | | -20 | | | 50 | | | | | | |  | |
| Left middle cingulate cortex |  |  | | 3.31 | | | -12 | | | -36 | | | 54 | | | | | | |  | |
| Left precentral gyrus (areas 3, 4) | 230 | .654 | | 3.58 | | | -30 | | | -30 | | | 60 | | | | | | |  | |
| Left inferior parietal lobe (angular gyrus) | 153 | .654 | | 3.36 | | | -46 | | | -68 | | | 26 | | | | | | |  | |
| Left middle occipital gyrus |  |  | | 2.98 | | | -34 | | | -78 | | | 30 | | | | | | |  | |
| Left intraparietal sulcus (hIP2) | 113 | .654 | | 2.99 | | | -50 | | | -36 | | | 42 | | | | | | |  | |
| Right occipital lobe (cuneus) | 58 | .654 | | 3.1 | | | 12 | | | -72 | | | 26 | | | | | | |  | |
| Right postcentral gyrus (area 6) | 43 | .654 | | 2.81 | | | 32 | | | -30 | | | 66 | | | | | | |  | |
|  |  |  | |  | | |  | | |  | | |  | | | | | | |  | |
| *(Neutral reasoning – neutral baseline) –*  *(Positive reasoning – positive baseline)* | | | | | | | | | | | | | | | | | | | | |  |
| Right fusiform gyrus | 38 | .873 | | 3.03 | | | 36 | | | -46 | | | -22 | | | | | | |  | |
|  |  |  | |  | | |  | | |  | | |  | | | | | | |  | |
| *(Negative reasoning – negative baseline) –*  *(Neutral reasoning – neutral baseline)* | | | | | | | | | | | | | | | | | | | | |  |
| Left postcentral gyrus (areas 3,2) | 799 | .089 | | | | 3.57 | | | -34 | | | -34 | | | | 58 | | | | | |
| Left postcentral gyrus (area 2) |  |  | | | | 3.52 | | | -44 | | | -32 | | | | 50 | | | | | |
| Left postcentral gyrus (areas 3,4) |  |  | | | | 3.44 | | | -38 | | | -22 | | | | 48 | | | | | |
| Right supplementary motor (areas 6,4) | 559 | .143 | | | | 4.68 | | | 6 | | | -22 | | | | 50 | | | | | |
| Left superior parietal (area 5) |  |  | | | | 3.57 | | | -8 | | | -30 | | | | 50 | | | | | |
| Left inferior frontal gyrus (triangularis) | 341 | .242 | | | | 3.76 | | | -40 | | | 24 | | | | 28 | | | | | |
| Left inferior frontal gyrus (triangularis) |  |  | | | | 3.44 | | | -38 | | | 10 | | | | 26 | | | | | |
| Right inferior parietal (angular gyrus) | 241 | .369 | | | | 3.83 | | | 46 | | | -64 | | | | 20 | | | | | |
| Left inferior parietal (angular gyrus) | 105 | .726 | | | | 3.16 | | | -44 | | | -68 | | | | 24 | | | | | |
| Left postcentral gyrus (OP4) | 95 | .726 | | | | 3.33 | | | -66 | | | -6 | | | | 18 | | | | | |
| Left inferior parietal (supramarginal gyrus) |  |  | | | | 3.01 | | | -54 | | | -20 | | | | 30 | | | | | |
| Left postcentral gyrus |  |  | | | | 2.89 | | | -66 | | | -14 | | | | 30 | | | | | |
|  |  |  | | | |  | | |  | | |  | | | |  | | | | | |
|  |  |  | | | |  | | |  | | |  | | | |  | | | | | |
| SI Table 2 *continued on next page ...* | | | | | | | | | | | | | | | | | | | | | |
| SI Table 2 *continued* | | | | | | | | | | | | | | | | | | | | | |
|  |  |  | | | |  | | |  | | |  | | | |  | | | | | |
| *(Neutral reasoning – neutral baseline) -*  *(Negative reasoning – negative baseline)* | | | | | | | | | | | | | | | | | | | | |  |
| Right hippocampus (EC, SUB) | 87 | .885 | | | | 3.22 | | | 22 | | | -6 | | | | -28 | | | | | |
|  |  |  | | | |  | | |  | | |  | | | |  | | | | | |
| *(Positive reasoning – positive baseline) –*  *(Negative reasoning – negative baseline)* | | | | | | | | | | | | | | | | | | | | |  |
| Right inferior frontal (orbitalis) | 428 | .264 | | | | 3.91 | | | 42 | | | 40 | | | | -14 | | | | | |
| Right superior parietal (area 7) | 36 | .924 | | | | 3.06 | | | 16 | | | -70 | | | | 52 | | | | | |
| Cerebellum (vermis) | 35 | .924 | | | | 2.9 | | | 0 | | | -56 | | | | -18 | | | | | |
| Left fusiform gyrus | 28 | .924 | | | | 3.06 | | | -34 | | | -6 | | | | -38 | | | | | |
|  |  |  | | | |  | | |  | | |  | | | |  | | | | | |
| *(Negative reasoning – negative baseline) –*  *(Positive reasoning – positive baseline)* | | | | | | | | | | | | | | | | | | | | |  |
| Left caudate nucleus | 594 | .131 | | | | 3.39 | | | -10 | | | 2 | | | | 20 | | | | | |
| Left inferior frontal gyrus (opercularis) |  |  | | | | 3.35 | | | -38 | | | 8 | | | | 26 | | | | | |
| Left occipital lobe (calcarine) | 47 | .936 | | | | 3.03 | | | -8 | | | -54 | | | | 6 | | | | | |
| Right middle temporal gyrus | 39 | .936 | | | | 2.86 | | | 44 | | | -62 | | | | 20 | | | | | |
| Right precentral gyrus (area 6) | 38 | .936 | | | | 2.85 | | | 48 | | | 0 | | | | 50 | | | | | |
| Right precentral gyrus (area 6) |  |  | | | | 2.78 | | | 58 | | | -2 | | | | 42 | | | | | |
|  |  |  | | | |  | | |  | | |  | | | |  | | | | | |
| *Conjunction:*  *(Positive reasoning – positive baseline) –*  *(Neutral reasoning – neutral baseline) and*  *(Negative reasoning – negative baseline) –*  *(Neutral reasoning – neutral baseline)* | | | | | | | | | | | | | | | | | | | | |  |
| Right supplementary motor (area 6) | 226 | .652 | | | | 3.34 | | | 6 | | | -20 | | | | 50 | | | | | |
| Left superior parietal (area 5) |  |  | | | | 3.24 | | | -10 | | | -34 | | | | 52 | | | | | |
| Left postcentral gyrus (areas 3,4) | 122 | .675 | | | | 3.43 | | | -32 | | | -32 | | | | 58 | | | | | |
| Left inferior parietal (angular gyrus) | 74 | .675 | | | | 3.16 | | | -44 | | | -68 | | | | 24 | | | | | |
| Left inferior parietal (intraparietal sulcus hIP2, supramarginal gyrus) | 34 | .675 | | | | 2.78 | | | -48 | | | -36 | | | | 46 | | | | | |

*^a^*Brain regions have been identified using SPM Anatomy Toolbox (Eickhoff et al., 2005)

*^b^*All reported regions are significant at p < .005 for 20 contiguous voxels uncorrected.

*^c^*At the Associate Editor’s request, we also provide the cluster *p*-value, corrected for multiple comparisons using FDR (Genovese et al., 2002).

Areas of peak activity include cluster size and are followed by other significant areas of activation in that cluster.

Appendix A: List of pictures used

set A

normed category IAPS system picture # trial#

positive 1650 1

neutral 2

negative 3000 3

negative 2493 4

positive 4180 5

neutral 6

neutral 7

positive 5628 8

negative 3140 9

neutral 10

positive 4320 11

neutral 12

positive 2058 13

positive 8030 14

neutral 15

positive 1460 16

neutral 17

negative 2200 18

neutral 19

negative 2410 20

negative 2280 21

positive 2070 22

neutral 23

neutral 24

negative 2499 25

positive 1811 26

neutral 27

negative 1230 28

neutral 29

negative 1050 30

positive 2050 31

negative 2230 32

negative 2221 33

positive 8031 34

neutral 35

neutral 36

positive 2057 37

negative 1120 38

positive 2040 39

negative 1935 40

Appendix A, *continued*

set B

normed category IAPS system picture # trial#

positive 2150 1

neutral 2

positive 4598 3

negative 3063 4

neutral 5

positive 4653 6

negative 3080 7

neutral 8

negative 2206 9

positive 1999 10

positive 2389 11

neutral 12

neutral 13

positive 4310 14

positive 2091 15

negative 2441 16

positive 4490 17

neutral 18

negative 3062 19

negative 3150 20

neutral 21

positive 4607 22

neutral 23

positive 4255 24

negative 7000 25

negative 7130 26

positive 4290 27

neutral 28

neutral 29

negative 3053 30

positive 1463 31

negative 2730 32

negative 3130 33

neutral 34

negative 3068 35

positive 2160 36

neutral 37

neutral 38

negative 3170 39

negative 7034 40

Appendix A, *continued*

set C

normed category IAPS system picture # trial#

negative 7160 1

negative 9080 2

neutral 3

positive 8041 4

negative 5130 5

negative 2491 6

positive 1722 7

negative 6260 8

negative 7036 9

neutral 10

neutral 11

neutral 12

negative 3069 13

positive 1610 14

positive 4220 15

negative 7038 16

positive 2209 17

neutral 18

positive 5621 19

positive 8021 20

neutral 21

neutral 22

neutral 23

positive 7289 24

positive 4660 25

negative 3030 26

neutral 27

negative 3120 28

neutral 29

positive 2080 30

neutral 31

positive 4530 32

positive 2165 33

positive 7580 34

negative 7037 35

neutral 36

neutral 37

negative 3064 38

negative 3010 39

positive 2057 40

Appendix B: Design Issues

**B 1. Event-related rather than block design, and ratings from participants rather than from the IAPS system.**

The pseudo-randomisation and the harvesting of intensity ratings (on the 8-point scale) were design choices to enhance any effect that the emotion manipulation might have on subsequent reasoning, with each exposure to emotion being a new and unpredicted event that the person would be experiencing. The individualized ratings incorporated not only intensity but valence in the same key press; in this way we harvested individualized valence ratings, reflecting our *a priori* choice to use actual effects of each valence on subsequent reasoning, rather than an effect predicted from group norms. For example, an individual participant rates a particular normed positive stimulus as neutral; if we were to classify that trial within the “positive” condition based on the normed valence, our positive-condition reasoning results would be made ‘noisy’ by this neutral response.

We were not concerned that there might be a transfer effect from the emotion of the previous trial, as there was the reasoning task, taking approximately 10 seconds, between each picture presentation. We thank the reviewers for raising concerns about such a transfer effect; the example was offered that a positive picture could be judged more positive after trials inducing negative emotion. To respond to this query, we conducted a paired-samples *t*-test, comparing the mean proportion of “high intensity: total” positive ratings that followed one-back positive trials (mean .206, *SD* .211), to those that followed one-back negative trials (mean .260, *SD* .269). The result was not significant (*t_13_ =-1.323, p* = .209).

**B 2. Modelling reasoning at the halfway point between conclusion and individual keypress and limiting ‘reasoning’ to the conclusion only, not the 2^nd^ premise.**

The task was to determine whether the conclusion follows logically from the premises. Expressed more formally, the task was to judge whether the argument is valid or not. It is first necessary to integrate the two premises and start to construct a representation of the problem; reasoning then takes place after the conclusion itself has been integrated into the problem to form an argument (Goel et al., 2000).

Other research in the field has focused on neural activation at each stage in this process; in the Goel opus (for example, Goel et al., 2000; Goel & Dolan, 2003a), the focus has been on this final reasoning component rather than on all stages of the process. As a consequence, neural results pertain to the period when argument validity is being assessed, but do not include sentence reading effects or activations associated with premise integration.

We avoid sentence reading effects by analysing reasoning timewindow data at the halfway point between the onset of the concluding sentence and the individual participant’s motor response (see for example Goel & Dolan, 2003a).

Appendix B, *continued*

**B 3. Possible effects of different emotions within the same valence.**

In the present study we distinguished between positive and negative valence conditions, but did not investigate the effects of specific emotions within each valence condition on reasoning. Elsewhere, it has been shown that specific emotions within the same valence condition, such as anger and fear, have differential effects on judgment and decision making: whereas fear promotes risk-averse choices, anger promotes risk-seeking choices (Lerner & Keltner, 2001). Furthermore, the divergent effects of fear and anger on risk perception are reliable, holding across experimentally induced and naturally occurring emotions (Lerner, Gonzalez, Small & Fischhoff, 2003). It would be worthwhile, in future studies, to explore the effect of particular emotions on syllogistic reasoning.

**B 4. Possible interaction of congruence (with beliefs) * emotional context.**

In the design of the current study, we controlled for the possible effect of belief-bias. Indeed, our manipulation check (see section 3.1.1) indicates that this control was necessary: correct responding was significantly hindered when the logic of the argument conflicted with beliefs.

Future studies could explore whether there is an effect at the neural level of (in)congruence * emotional context on reasoning. To ensure sufficient power, it may be prudent to limit such investigation to ‘neutral *versus* negative’ in one study, and ‘neutral *versus* positive’ in another.

Additionally, we could propose a study directly comparing the effects of belief-bias on reasoning about emotional content *versus* reasoning about neutral subject-matter in an emotional context.
